# Dual-mode aptamer-driven biosensing platform for ultrasensitive and mutation-resilient detection of the SARS-CoV-2 nucleocapsid protein

**DOI:** 10.1016/j.gendis.2025.101943

**Published:** 2025-11-19

**Authors:** Shu Zhou, Yuxi Xu, Huan Liao, Hailong Ou, Dan Qi, Yatao Wu, Yunyi Liu, Juan Li, Jiaxuan Li, Bi Shi, Fei Zhu, Siran Zhang, Jason H. Huang, Erxi Wu, Xiaoxiao Hu

**Affiliations:** a State Key Laboratory of Chemo and Biosensing, College of Biology, Molecular Science and Biomedicine Laboratory, Hunan Research Center of the Basic Discipline for Cell Signaling, Hunan University, Changsha, Hunan 410082, China; b Shenzhen Research Institute, Hunan University, Shenzhen, Guangdong 518000, China; c Innovation Institute of Industrial Design and Machine Intelligence, Quanzhou-Hunan University, Quanzhou, Fujian 362006, China; d Research Institute of Hunan University in Chongqing, Chongqing 401120, China; e Department of Neurosurgery, Neuroscience Institute, Baylor Scott & White Health, Temple, TX 76508, USA; f Department of Neurosurgery, Baylor College of Medicine, Temple, TX 76502, USA; g Texas A&M University Colleges of Medicine and Pharmacy, College Station, TX 77843, USA; h Livestrong Cancer Institutes, Department of Internal Medicine, Dell Medical School, The University of Texas at Austin, Austin, TX 78712, USA

**Keywords:** Aptamer, Aptamer-antibody sandwich assay, Dynamic light scattering, Mutation, SARS-CoV-2 N protein

## Abstract

Severe acute respiratory syndrome coronavirus 2 (SARS-CoV-2) remains a significant global health threat because of its rapid evolution and high mutation rate, which limits the performance of existing molecular diagnostics. This study presents a dual-mode, aptamer-based detection platform that combines high sensitivity with mutation resilience. Using a computer-assisted X-aptamer Systematic Evolution of Ligands by EXponential enrichment (SELEX) approach, we identified NP14, a high-affinity, dual-target DNA aptamer that specifically binds to the SARS-CoV-2 nucleocapsid (N) protein at its N-terminal domain. Analyses via molecular docking, aptamer truncation, and targeted mutagenesis revealed that NP14 interacted with both SARS-CoV-2 and SARS-CoV N proteins and identified key nucleotides C24 and G27 of the P1 region and structural determinants critical for its high-affinity binding. Building on this discovery, we engineered a dual-mode biosensing system by integrating NP14 into a multicolor dynamic light scattering-enhanced enzyme-linked aptamer-antibody assay (MD ELAAA). MD ELAAA synergistically combines two complementary detection strategies: i) non-aggregative plasmonic colorimetry for visual signal detection and ii) dynamic light scattering for ultrasensitive quantitative analysis, in which Au/Ag nanomaterials are used to amplify optical and scattering signals. This system achieves a sensitivity of 0.43 TCID_50_/mL, representing a 47-fold improvement over standard methods. By integrating high sensitivity, specificity, variant recognition, and dual-mode signal output, the MD ELAAA platform enables reliable detection of low-abundance SARS-CoV-2 antigens. Its robust performance supports early-stage diagnostics and high-throughput variant monitoring, establishing MD ELAAA as a robust platform for next-generation viral detection and surveillance.

## Introduction

The emergence of coronavirus disease 2019 (COVID-19) has posed significant risks to immunocompromised and elderly individuals, but the persistent evolution of severe acute respiratory syndrome coronavirus 2 (SARS-CoV-2) variants and subvariants remains a critical concern. These variants, characterized by frequent mutations, can alter the virus’s infectivity, transmissibility, and antigenicity, further complicating disease control efforts. Therefore, real-time monitoring of variant spread is crucial for mitigating this ongoing threat. This urgent need underscores the importance of developing a rapid, intuitive, highly sensitive, and specific detection technology capable of identifying SARS-CoV-2 variants. Among potential diagnostic targets, the SARS-CoV-2 N protein is essential for viral replication and immune system interactions, making it a key biomarker for variant detection.^1^, ^2^, ^3^ The detection of the SARS-CoV-2 N protein has been achieved through multiple analytical platforms, such as reverse transcription PCR^4^^,^^5^ and immunoassays.^6^^,^^7^ Among these methods, immunoassays have emerged as a well-established approach because of their high sensitivity and specificity, rapid turnaround times, and compatibility with high-throughput screening.^8^^,^^9^ Furthermore, their cost-effectiveness and operational simplicity make them particularly suitable for large-scale deployment in resource-limited settings. The conventional double-antibody sandwich enzyme-linked immunosorbent assay (ELISA) is a well-established, rapid, and highly selective immunoassay method that relies on specific antibody pairings to detect large proteins.^10^ However, its effectiveness is limited when detecting small proteins because of steric hindrance between antibodies, which compromises binding efficiency.

To overcome this limitation, aptamer-nucleic acid sequences capable of binding with high specificity to low-molecular-weight compounds offer a superior alternative owing to their structural flexibility and ability to recognize small targets with minimal steric interference.^11^ Luo et al rapidly screened aptamers for N protein^12^ and demonstrated that the aptamer-antibody sandwich method was more sensitive than the double-antibody or double-aptamer sandwich method for the detection of N protein.^13^ Although DNA aptamers that bind to the SARS-CoV-2 N protein have been demonstrated in earlier studies,^12^^,^^14^, ^15^, ^16^, ^17^ few studies have investigated the mechanism of SARS-CoV-2 N recognition by aptamers. A deeper understanding of aptamer binding mechanisms, including the selection of high-affinity aptamers and structural determinants of target recognition, is critical for improving diagnostic reliability. Previous studies have reported the binding mechanism of aptamer A58, which targets the N-terminal domain (NTD) of the SARS-CoV-2 N protein. A58 is a compact stem-loop DNA aptamer whose hexanucleotide DNA motif (5′-TCGGAT-3′) plays a critical role in binding, with T6 showing the most significant impact on binding activity.^18^ However, aptamers with different sequences and secondary structures have different binding mechanisms to target proteins.^19^ Moreover, the integration of the aptamer-antibody mode in ELAAA enhances both sensitivity and specificity, overcoming the limitations associated with conventional biomolecules while also enabling the simultaneous detection of multiple targets.^16^ Despite its promise, the visual detection capabilities of conventional ELAAA are constrained by its monochromatic response, making it difficult to distinguish with the naked eye. Furthermore, its detection limit remains confined to the nanomolar range.^20^^,^^21^

Advances in nanotechnology have facilitated the introduction of noble metal nanoparticles, such as gold and silver (Au/AgNPs), which exhibit high molar extinction coefficients and exceptional surface plasmon resonance (SPR) properties. These unique optical characteristics enable multicolor sensing, making noble metal nanoparticles ideal for applications in medical diagnostics and food safety.^22^, ^23^, ^24^, ^25^ Conventional metal nanoparticle-based methods often rely on aggregation-dependent sensing, which can be problematic in complex samples because of spontaneous auto-aggregation, leading to inconsistent and unreliable results. To address this challenge, recent research has focused on “non-aggregation” strategies that mitigate these issues and generate clear, distinct color changes, improving diagnostic reliability.^26^, ^27^, ^28^ Owing to their numerous advantages, including facile synthesis, outstanding biocompatibility, high specific surface area, and strong extinction coefficients, gold nanoflowers (AuNFs) offer a highly effective platform for straightforward visual interpretation. Specifically, AuNFs have been implemented in a novel non-aggregated multicolor detection strategy, leveraging their superior colorimetric properties to improve accuracy and sensitivity.^23^^,^^29^ Research has demonstrated that AuNF-based colorimetric biosensors can effectively detect a variety of biological targets, including mitochondrial toxins, mycotoxins,^30^ natural compounds,^31^ and cancer biomarkers.^32^ Previous studies have demonstrated that integrating the plasmonic signal changes induced by the growth of AuNFs with ELISA can increase the sensitivity by an order of magnitude.^33^ However, the visual readout of colorimetric biosensors remains confined to qualitative or semi-quantitative analysis based on optical absorption signals, which fails to meet the requirements for ultrasensitive detection of trace-level biomarkers, thereby significantly limiting their broader diagnostic applications.

In addition to colorimetric analysis, the incorporation of dynamic light scattering (DLS) into ELISA significantly enhances its quantitative capabilities. Owing to strong SPR effects, the light scattering intensity of noble metal nanoparticles is 2–3 orders of magnitude greater than that of polymer beads of similar size, enabling highly sensitive signal detection and minimizing background interference from complex biological matrices.^34^^,^^35^ Owing to the ultra-high sensitivity of light scattering signals, numerous light scattering-based sensors have been developed for the detection of various targets, including mycotoxins,^35^ viruses,^36^ proteins,^37^^,^^38^ and microorganisms.^39^ In these sensors, the changes in light scattering signals induced by target-mediated growth or etching of AuNFs represent a commonly employed strategy. Tong et al established AuNP etching combined with light scattering signal-mediated multicolor ELISA for staphylococcal enterotoxin detection.^40^ However, the strategy of multicolor ELISA coupled with light scattering signals is still limited to the antibody system and fails to fully utilize the advantages of the aptamer-antibody model.

Here, we obtained an aptamer that targets the SARS-CoV-2 N protein, named NP14, based on an X-Aptamer protein screening strategy, and then developed an aptamer-antibody sandwich mode-mediated MD ELAAA method for high-sensitivity detection of the N protein of SARS-CoV-2. The obtained NP14 recognized several variants of SARS-CoV-2, including alpha, beta, gamma, delta, lambda, and omicron variants. Notably, this detection strategy not only uses aptamers for target recognition but also further reveals the dominant role of G and C bases in interface recognition through molecular docking, truncation, and targeted mutagenesis experiments. Compared with previous aptamers, NP14 has better affinity for NTD protein binding, which broadens new ideas for the binding mechanism of SARS-CoV-2 N protein aptamers and improves the detection limit of the whole detection process. The diagnostic system can detect 0.39 pg/mL SARS-CoV-2 N protein with high throughput at room temperature. Finally, we detected the virus cultures in the lysate spiked at 0.43 TCID_50_/mL (median tissue culture infectious dose). Compared with previously reported aptamer-based assays, this method has the advantages of a lower detection limit, high throughput, naked-eye semi**-**quantification, and single-factor dual-signal output.

## Methods

### Materials and reagents

DNA aptamers were synthesized by General Biotechnology (Anhui, China). The sequences are displayed in Table S1. PCR Primer Synthesis from Tsingke Biological Technology. Chloroauric acid (HAuCl_4_·3H_2_O), hydroquinone, silver nitrate (AgNO_3_), and trisodium citrate (Na_3_C_6_H_5_O_7_·2H_2_O) were purchased from Sigma–Aldrich (St. Louis, Missouri, USA). Bovine serum albumin (BSA) was obtained from BioFroxx (Guangzhou Sego Bio Co. Ltd.). X-Aptamer libraries were acquired from AM Biotechnologies (Houston, Texas, USA); His-Tag magnetic beads (Invitrogen, Dynabeads™ His-Tag Isolation & Pulldown, 10103D), SARS-CoV-2 N protein, and anti-SARS-CoV-2 N protein monoclonal antibodies (anti-SARS-CoV-2 N protein mAb, Cat: 40143-MM05, 40588-R001) were purchased from Sino Biological. Inc (Beijing, China). Ultrapure water was obtained from a Milli-Q A facility (Molsheim, France). The 96-well microtiter plates and streptavidin-horseradish peroxidase (SA-HRP) were purchased from Sangyo Biotech (Shanghai, China). TMB (3,3′,5,5' - tetramethylbenzidine) substrate and stop solution were purchased from Beijing Solare Bio-Technology Co., Ltd., Beijing, China. Dulbecco's phosphate-buffered saline (DPBS), phosphate-buffered saline (PBS), nuclease-free water, Tween 20, and universal dilutions were obtained from Sevier Biotechnology Co., Ltd., Wuhan, China. NaH_2_PO_4_·H_2_O, His labeling gel strain, streptavidin-coupled alkaline phosphatase (ALP) (SA-ALP), and imidazole were procured from Beyotime Biotechnology Co., Ltd., Changsha, China. l-ascorbic acid (L-AA) and l-ascorbic acid-2-phosphate trisodium salt (AAP) were obtained from Macklin Corporation (Shanghai, China). Lysates and mutant proteins were acquired from Fipon (Dongguan, China). A BCA kit and Anti-Flag MagBeads (Cat: 20565ES03) were obtained from Yeasen Corporation (Shanghai, China). Fetal bovine serum (FBS) was acquired from ABW-Bio (Shanghai, China). SARS-CoV-2 N protein-receptive bacteria were obtained from Guangdong Provincial Biotechnology Research Institute. The SARS-CoV N protein, HCoVs229E, HCoVs-OC43, HCoVs-HKU1, and ELISA kits (Cat: CSB-EL33251, https://www.cusabio.com/) were purchased from CUSABIO (Wuhan, China). Equilibrium filtration columns were obtained from ProMab Biotechnology (Changsha, China). The protein marker (RM19001) was purchased from ABclonal Biotechnology Co., Ltd., Wuhan, China. The DNA loading buffer was purchased from Diagbio Biotechnology Co., Ltd., Wuhan, China. Cell counting was performed using Cell Countstar Rigel S2 Fluorescence Cell Analyzer (ALIT Life Science Co., Ltd.). Plasmid extraction kit (Reagent No. DC204) from Vazyme Biotech Co., Ltd., Nanjing, China. Serum-free cell freezing medium (Cat: C40100) was supplied by New Cell & Molecular Biotech. Cell culture dishes were provided by BaiDi Biotechnology Co., Ltd. (BDBIO). Antibody dilution buffer (Cat: PS114) was supplied by Epizyme (Shanghai, China). The confocal dish was purchased from NEST Biotechnology Co., Ltd., Wuxi, China. Dulbecco's modified Eagle medium (DMEM) was provided by Procell Life Science&Technology Co., Ltd.

### Apparatus

Transmission electron microscopy and energy dispersive spectrometer data analysis were supported by Ceshigo Research Service (www.ceshigo.com). UV–visible absorption spectral analysis was conducted using a UV–visible spectrophotometer (Shimadzu, Japan). A confocal laser scanning microscope was obtained from OLYMPUS (Japan). Flow cytometry was from BD Biosciences (Shanghai, China). The Universal Microplate Spectrophotometer was obtained from Thermo Fisher (Thermo Fisher, USA). DNA gel electrophoresis results were obtained via a ChemiDoc™ XRS Imager (Bio-Rad, USA). The DLS values of the AuNFs and AuNF@Ag were measured via a Malvern Zetasizer Nano ZS90 system (Malvern Instruments Ltd, Worcestershire, UK). CD spectroscopy was obtained from JASCO (Japan).

### X-aptamer protein SELEX

The initial library was first incubated with His-tag magnetic beads for negative selection, and the microsphere library that was magnetically separated and retained after incubation was used as the negative selection sample. The aptamer microspheres that did not bind to the magnetic beads were collected and then incubated with magnetic beads encapsulating His-SARS-CoV-2 N protein, and the library of aptamer microspheres bound to the magnetic beads was magnetically separated and collected. The target aptamer enriched from the first round of screening was released from the microspheres through alkaline cleavage and collected via equilibrium filtration columns, resulting in the first round of positive screening. Positive products were subsequently amplified through PCR and column purification.

### Preparation of the recombinant His-SARS-CoV-2 N protein

First, the activated bacterial mixture was inoculated into 4 mL of kanamycin-resistant LB culture medium at a ratio of 1:10 (bacterial species: medium) and placed on a shaker at 37 °C and 170 rpm for 3–4 h. Then, the inoculum was transferred to 100 mL of kanamycin-resistant LB culture medium at a ratio of 1:100 (bacterial species: medium) and placed on a shaker at 37 °C and 170 rpm. One hundred milliliters of kanamycin-resistant LB culture medium was inoculated at a ratio of 1:100 (strain: medium), placed on a shaker at 37 °C and 170 rpm, and incubated until the OD_600_ reached 0.6–0.8. The inducer IPTG was subsequently added to a final concentration of 1 mM, and the culture was placed on a shaker at 37 °C and 170 rpm, and incubated overnight. The organisms were then collected by centrifugation and resuspended in lysate to a volume of 10 mL. Next, the *E. coli* was lysed via a cell crusher, the bacterial precipitate was collected, and the proteins were purified via His-tag purification resin. Finally, the concentration of the N protein was determined via a BCA kit and stored at −80 °C for subsequent experiments.

### SPR analysis

Studies were conducted using a Biacore T200 (GE Healthcare) machine at 25 °C with CM5 chips. Next, wash buffer (PBSTM, 0.01 M, pH 7.4) was injected at a relatively high flow rate to flush the entire system within the flow path using a wash buffer pipette tip. N-hydroxysuccinimide (NHS) and 1-(3-dimethylaminopropyl)-3-ethylcarbodiimide (EDC) were subsequently injected to activate the dextran carboxylate on the surface of the CM5 chips. Ligand proteins were then injected into a low-pH sodium acetate buffer to ensure that a sufficient amount of protein was present on the dextran. All carboxylic acid esters were treated without reaction activation using ethanolamine as a blocking solution. Afterwards, the first 50 candidate sequences (500 nM, 200 μL) were detected individually.

Based on the screening results of the previous 50 DNA aptamers, the NP14 aptamer was selected for multi-gradient testing. Various concentrations of NP14 (15.625, 31.25, 62.5, 125, 250, and 500 nM) were detected separately. The analyte binding time was set to 180 s at a flow rate of 20 μL/min; the dissociation time was 240 s at a flow rate of 20 μL/min; and the regeneration time was 30 s at a flow rate of 20 μL/min. Finally, the separation and binding rate constants were calculated via BIA evaluation software at the point–click interface.

### Flow cytometry analysis

To monitor the enrichment of selected libraries or determine the binding affinity of specific aptamers, positive SARS-CoV-2 N protein-His tag beads were incubated with 300 nM 5′-FAM labeled NP14 or the library, in 300 μL of binding buffer at 4 °C for 30 min. The beads were subsequently washed twice with binding buffer and resuspended in 200 μL of binding buffer. The fluorescence intensity of the beads was measured via flow cytometry by counting approximately 10,000 events.

### Confocal microscopy imaging

Three microliters of 12.5 mg/mL diluted His purification magnetic beads were washed twice with 500 μL of DPBS and incubated with 1 μg of His-tagged target protein for 30 min at room temperature with rotation. The bead-protein complex was washed twice with 500 μL of DPBS to remove unbound protein and then resuspended in 200 μL of DPBS. The 300 nM of 5′-FAM-labeled library or NP14 was incubated with the complex of magnetic beads and His-tagged protein at room temperature for 30 min. After washing twice with 500 μL of DPBS, the complex was finally resuspended in 200 μL of DPBS and transferred to a glass slide for fluorescence imaging. The fluorescence image was collected via both the green fluorescence and transmitted light channels via confocal microscopy.

### Aptamer stability

The stability of NP14 in 10% FBS was analyzed via DNA PAGE gel. Subsequently, 100 μL of 5′-FAM-labeled NP14 (3 μM) was added to 10% FBS and incubated at 37 °C for 0 h, 1 h, 2 h, 4 h, 8 h, 12 h, 24 h, 36 h, 48 h, or 72 h. At the specified time points, the samples were denatured at 100 °C for 5 min and then stored at −80 °C. All the samples were analyzed via 12% DNA PAGE.

### Structural prediction of NP14 and its molecular docking with the SARS-CoV-2 N protein

The structures of the SARS-CoV-2 N proteins were obtained from the RCSB PDB database (http://www.rcsb.org,ID:6VYO). The free energy-minimized secondary structures of the aptamer were predicted via the Nupack web server. The 3D structure of the nucleocapsid aptamer was subsequently generated in the RNA composer by replacing thymine (T) with uracil (U), and the corresponding 3D structure of the equivalent single-stranded RNA (ssRNA) was modeled and visualized. During the preliminary docking process, a total of 100 protein-aptamer interaction phases were generated; the conformation with the highest probability was selected for the final simulation, and the conformations with the lowest docking energy levels were organized for analysis.

### Antibody–antibody sandwich enzyme‒linked immunosorbent assay (ELISA) platform

Briefly, anti-SARS-CoV-2 N protein monoclonal antibody (mAb) microtiter plates were fixed overnight at 4 °C, washed three times with 0.05% PBST, and blocked with 5% BSA for 1 h. After washing, another pair of antibodies was added and incubated at 37 °C for 1 h. Subsequently, anti-mouse IgG coupled to horseradish peroxidase was added and incubated at 37 °C for 0.5 h. After washing, 100 μL of a single-component chromogenic solution was added for the chromogenic reaction, which was subsequently stopped by the addition of 50 μL of termination solution. Finally, the average optical density (OD) was measured via a Universal Microplate Spectrophotometer.

### Enzyme-linked oligonucleotide assay (ELONA)

Different concentrations of SARS-CoV-2 N protein (0, 0.5, 1, 5, 10, 20, 50, 100, 200, 500, or 1000 ng/mL) were immobilized in the microwell plate overnight at 4 °C, followed by three washes with 0.05% PBST and blocking with 5% BSA for 1 h. After washing, the NP14 aptamer was added, and the mixture was incubated at 37 °C for 1 h. Subsequently, streptavidin-coupled horseradish peroxidase (HRP) (SA-HRP) was added, and the mixture was incubated at 37 °C for 0.5 h. Following washing, 100 μL of a single-component chromogenic solution was added for the chromogenic reaction, which was subsequently terminated by the addition of 50 μL of termination solution. Finally, the average optical density (OD) was measured using a Universal Microplate Spectrophotometer.

### Enzyme-linked aptamer-antibody sandwich assay (ELAAA)

Briefly, anti-SARS-CoV-2 N protein microtiter plates coated with monoclonal antibody (mAb) were fixed overnight at 4 °C, followed by three washes with 0.05% PBST and blocking with 5% BSA for 1 h. After washing, biotin-coupled NP14 was added and incubated at 37 °C for 1 h. Subsequently, streptavidin-horseradish peroxidase (SA-HRP) was added, and the mixture was incubated at 37 °C for 0.5 h. After washing, 100 μL of single-component chromogenic solution was added for the chromogenic reaction, which was subsequently terminated by adding 50 μL of termination solution. Finally, the average optical density was measured via a universal microplate spectrophotometer.

Furthermore, we conducted a checkerboard optimization of the mAb and aptamer concentrations, as illustrated in Table S2. For subsequent experiments, we selected an aptamer concentration of 200 nM and an antibody concentration of 1 μg/mL as the optimal experimental parameters for ELAAA.

### Dual-mode MD ELAAA experiment

Anti-SARS-CoV-2 N protein mAb (1 μg/mL) microtiter plates were fixed at 4 °C overnight, washed three times with 0.05% PBST, and blocked with 5% BSA for 1 h. Following the washing step, biotin-coupled NP14 was added and incubated at 37 °C for 1 h. Subsequently, SA-ALP was added, and the mixture was incubated at 37 °C for 0.5 h. After washing, 100 μL of AAP was added, and the mixture was incubated at room temperature for 0.5 h. Finally, 35 μL of a mixture of AuNFs and AgNO_3_ was added to the mixture for color development at 37 °C for 0.5–1 h. DLS was then used to measure the light scattering intensity.

### Plasmid generation and transfection

To express Flag-SARS-CoV-2 N1-ZS Green in mammalian cells, HEK293 cells were plated at a density of 3 × 10^6^ in a 10 cm culture dish and allowed to adhere overnight. The next day, the cells were transfected with 15 μg of plasmid and 24 μL of Lipo8000 transfection reagent. The Lipo8000–plasmid complex was prepared in serum-free DMEM and then added to the cells in fresh DMEM containing 10% FBS. After 24 h of transfection, the medium was replaced with fresh DMEM supplemented with 10% FBS, and the cells were cultured for an additional 24 h. Fluorescence microscopy was then used to verify plasmid transfection and expression. The cells were subsequently lysed using RIPA buffer to extract total protein, followed by centrifugation at 12,000 *g* for 15 min to retain the supernatant, which was stored at −80 °C for future use.

### Circular dichroism spectroscopy characterization

Using 300 μL of PBS as a blank control, 300 μL of unlabeled AS1411 (20 μM) or NP14 (10 μM) was taken and measured using a circular dichroism spectrometer.

### Statistical analysis

DNA gel was processed with ImageJ to obtain grayscale values, and analyzed via SigmaPlot (v. 12.5) to create the plots. Experimental results were presented as mean ± standard deviation. Variance analysis was performed using GraphPad Prism (v. 9.0). Statistical evaluations were conducted using a two-tailed Student's *t*-test, one-way and two-way analysis of variance, with *p* < 0.05 considered statistically significant (∗*p* < 0.05, ∗∗*p* < 0.01, ∗∗∗*p* < 0.001, and ∗∗∗∗*p* < 0.0001).

## Results

### Identification of NP14 via X-aptamer SELEX

**Figure 1:**
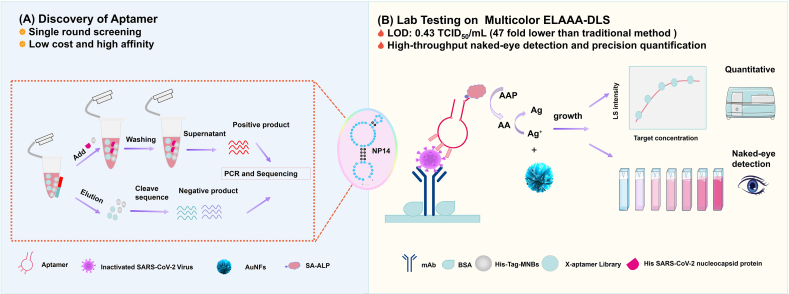
Workflow of NP14 aptamer screening and development of the MD ELAAA detection platform. **(A)** Schematic illustration of the X-aptamer protein SELEX process for isolating aptamers. **(B)** Schematic illustration of the ultrasensitive detection of the SARS-CoV-2 N protein via the MD ELAAA platform.

To isolate high-affinity aptamers against the SARS-CoV-2 nucleocapsid (N) protein, we employed an X-aptamer-based SELEX strategy (Fig. 1A) using a random single-stranded DNA (ssDNA) library provided by AM Biotechnologies (Houston, USA). The selection process began with a negative selection step in which His-tag-coated magnetic beads were pre-blocked with BSA to remove sequences with nonspecific binding.^41^ This step eliminated the aptamers that bound non-specifically to the magnetic beads or were considered false positives. The negatively selected library was then incubated with magnetic beads coated with His-tagged SARS-CoV-2 N protein to enrich target-specific binders, forming the positive selection library. The enriched X-aptamer oligonucleotides were amplified via PCR. To optimize the yield while minimizing nonspecific amplification, 30 PCR cycles per round were chosen as the optimal condition (Fig. S1). Excessive cycling can result in byproducts that compromise the accuracy and efficiency of downstream cloning and sequencing. The resulting DNA pools were then subjected to high-throughput sequencing. We used MAGE7 software for multiple sequence alignment and phylogenetic tree analysis, classified aptamer sequences and homology, and selected representative candidate sequences.^41^^,^^42^ Based on the abundance and preliminary binding estimates to the N protein, 50 candidate aptamers were selected for further evaluation (Fig. S2A). These candidates were assessed using SPR to determine their binding affinities for the SARS-CoV-2 N protein (Fig. S2B). Among the tested sequences, three aptamers—NP1, NP11, and NP14—showed high binding responses and sequence conservation. Of these, NP14, a 52-nucleotide DNA aptamer (sequence in Table S1), exhibited the strongest and most consistent binding characteristics (Fig. S3). Consequently, NP14 was selected as the lead candidate for downstream structural and functional characterization. In conclusion, through iterative selection, phylogenetic clustering, and SPR validation, NP14 was identified as a 52-nucleotide aptamer with high binding affinity for the SARS-CoV-2 N protein.

### Binding affinity, thermostability, and serum resistance of NP14

**Figure 2:**
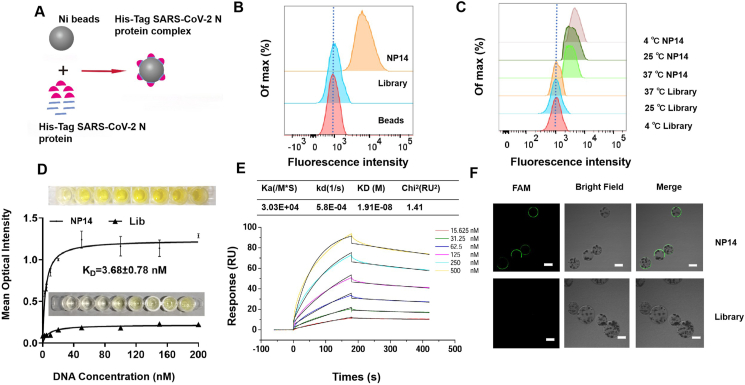
Binding affinity and stability characterization of the NP14 aptamer. **(A)** Magnetic bead (12.5 mg/mL, 3 μL) flow assay for the binding of the aptamer to the His-tag SARS-CoV-2 N protein (1 μg). **(B)** Flow cytometry analysis of the binding of 300 nM FAM-labeled aptamer NP14 to magnetic beads coated with the SARS-CoV-2 N protein. **(C)** Flow cytometry analysis of the binding of 300 nM FAM-labeled NP14 to magnetic beads coated with the SARS-CoV-2 N protein at different temperatures (4 °C, 25 °C, and 37 °C). **(D)** The binding affinity of NP14 for the SARS-CoV-2 N protein was validated via the use of 2 μg/mL SARS-CoV-2 N protein and biotin-labeled NP14 at different concentrations (0, 2.5, 5, 10, 20, 50, 100, 150, and 200 nM). **(E)** Determination of the Kd value of aptamer NP14 (15.625, 31.25, 62.5, 125, 250, and 500 nM) via surface plasmon resonance. **(F)** Confocal analysis of 300 nM FAM-labeled aptamer NP14 with SARS-CoV-2 N protein-coated magnetic beads (scale bar = 30 μm).

To evaluate the binding performance of the selected aptamer, the recombinant SARS-CoV-2 N protein was successfully expressed and purified. SDS-PAGE analysis confirmed the presence of the 48 kDa recombinant protein in the elution fractions (Fig. S4A). ELISA assays further verified the immunogenicity of the purified N protein, which was strongly reactive with paired antibodies compared with the control groups (Fig. S4B). The individual synthetic aptamers were then incubated with the viral mimics at room temperature, and binding was assessed by flow cytometry (Fig. 2A). Flow cytometry analysis revealed that aptamer NP14 exhibited significantly higher binding affinity than the initial library did (Fig. 2B), which was further corroborated by confocal microscopy (Fig. 2F). Notably, NP14 maintained robust binding to the SARS-CoV-2 N protein across a broad temperature range (4 °C, 25 °C, and 37 °C), suggesting its adaptability under various experimental conditions (Fig. 2C). The dissociation constant (K_D_) of NP14 as determined by an enzyme-linked oligonucleotide assay (ELONA) was 3.68 nM, confirming its high binding affinity (Fig. 2D) relative to that of NP1 and NP11 (Fig. S5). SPR analyses validated and confirmed the binding kinetics of NP14 with the SARS-CoV-2 N protein (Fig. 2E), providing precise kinetic parameters (KD, ka, kd), with the KD value indicating high binding affinity. These results corroborate the ELONA-derived affinity values (K_D_ = 3.68 nM; Fig. 2D), reinforcing the robustness of the NP14 binding measurements and the usefulness of ELONA for assessing aptamer binding profiles. In addition to its affinity, NP14 exhibited excellent serum stability, maintaining structural integrity for up to 18 h in serum-containing conditions (Fig. S6A and S6B). These findings collectively establish NP14 as a highly stable and potent aptamer candidate for sensitive and robust detection of the SARS-CoV-2 N protein.

### Structural characterization of NP14–N protein binding interactions

**Figure 3:**
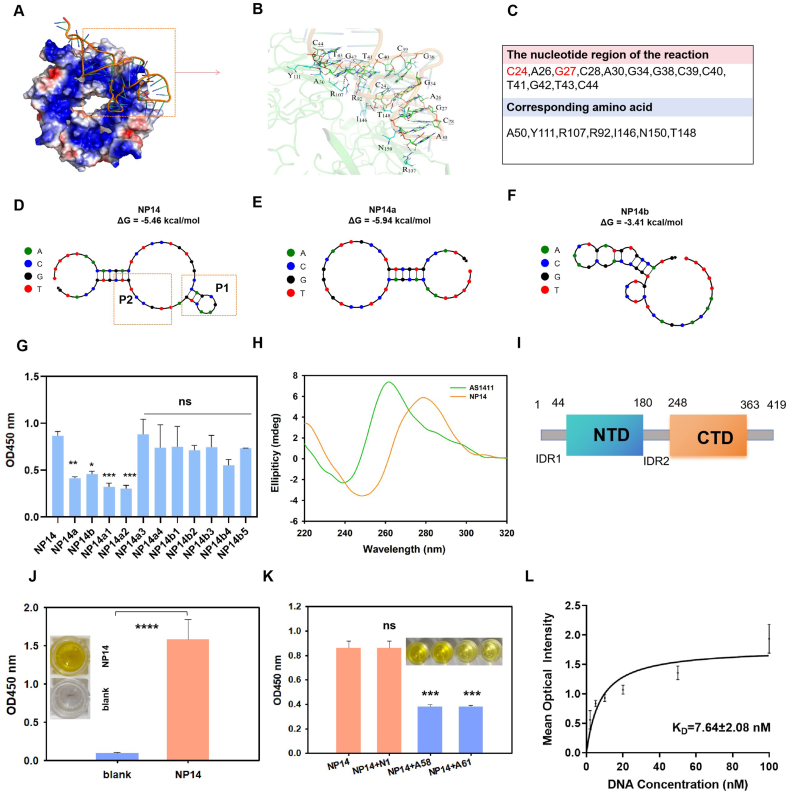
Structural basis and binding mechanism of NP14 interaction with the SARS-CoV-2 N protein. **(A)** Molecular simulation of the binding mode between aptamer NP14 and the SARS-CoV-2 N protein (http://www.rcsb.org, ID:6VYO) via AutoDock. **(B)** Enlarged view of the presumed binding area. **(C)** Nucleic acid sequences and corresponding amino acids involved in the docking model. **(D)** Secondary structure simulation of aptamer NP14 via the Nupack web server at 37 °C. **(E)** Secondary structure simulation of the truncated chains NP14a via the Nupack web server at 37 °C. **(F)** Secondary structure simulation of the truncated chains NP14b via the Nupack web server at 37 °C. **(G)** Binding analysis of NP14 with truncated NP14a, NP14b, and base-mutated 400 nM NP14a1, NP14a2, NP14a3, NP14a4, NP14b1, NP14b2, NP14b3, NP14b4, and NP14b5 to the SARS-CoV-2 N protein by ELONA. Data were presented as mean ± standard deviation of triplicate results (*n* = 3). The NP14 control: ns, not significant; ∗*p* < 0.05, ∗∗*p* < 0.01, and ∗∗∗*p* < 0.001. **(H)** Circular dichroism spectroscopy of AS1411 (20 μM) and NP14 (10 μM) was performed in PBS buffer (0.01 M, pH = 7.4) at wavelengths ranging from 220 to 320 nm. **(I)** Domain organization of the SARS-CoV-2 N protein, with numbers indicating domain boundaries. **(J)** Immunomagnetic beads (40 μL, 10 mg/mL) labeled with Flag antibodies against the truncated overexpressed protein were reacted with 300 nM biotin-labeled NP14 to assess binding. Data were presented as mean ± standard deviation of triplicate results (*n* = 3). Compared with the blank control: ∗∗∗∗*p* < 0.0001. **(K)** 250 nM biotin-labeled NP14 was mixed with 250 nM unlabeled N1, A58, A61 and competitive binding was analyzed by ELONA. Data were presented as mean ± standard deviation of four replicate results (*n* = 4). Compared with the NP14: ns, not significant; ∗∗∗*p* < 0.001. **(L)** Evaluation of the binding affinity for truncated proteins containing the NTD region at different concentrations of NP14 (0, 2, 5, 10, 20, 50, and 100 nM). Data were presented as mean ± standard deviation of triplicate results (*n* = 3).

After confirming that the aptamer NP14 has a strong affinity for SARS-CoV-2 N, the next logical step is to investigate the structural characteristics of NP14 and its binding mechanism with SARS-CoV-2 N. First, to clarify the binding site and interaction mode between NP14 and the SARS-CoV-2 N protein, we conducted semiflexible molecular docking using AutoDock software, with the receptor proteins being rigid and the aptamers being flexible. Then, we evaluated the docking results by calculating the binding free energy.^43^ During the docking process, we extracted 100 components and initiated docking. We ranked the energy values and utilized the minimum energy conformer to predict the binding site between the protein and the aptamer. Using PyMOL software, we further analyzed the construct, searched for amino acid residues within 4 Å of NP14, and highlighted the presence of hydrogen bonds with a black dotted line (Fig. 3A). In detail, as shown in Figure 3B and C, the C24, A26, G27, C28, A30, G34, G38, C39, C40, T41, G42, T43, and C44 nucleotide regions of the aptamer NP14 are within 4 Å of the amino acid residues alanine (A) 50, arginine (R) 92, R107, tyrosine (Y) 111, isoleucine (I) 146, threonine (T) 148, and asparagine (N) 150 of the SARS-CoV-2 N protein, suggesting that NP14 may bind to the SARS-CoV-2 NTD. Aptamers achieve high affinity and specific recognition of targets such as proteins and small molecules by folding into specific three-dimensional structures.^44^ To further understand its structural basis in performing complex functions, we analyzed the secondary structure of NP14 according to the molecular docking results and found that it consists of two paired regions, P1 (C24-G34) and P2 (T36-C44) (Fig. 3D). We subsequently truncated the two paired regions P1 and P2, named NP14a and NP14b, respectively, and the secondary structure is shown in Figure 3E and F. The binding properties of NP14a and NP14b to SARS-CoV-2-N proteins were subsequently evaluated via ELONA. The results of Figure 3G show that both NP14a and NP14b cause a decrease in binding activity, with NP14a causing a greater loss of binding activity, indicating that the P1 and P2 regions of NP14 are essential for binding SARS-CoV-2 and that important nucleotide elements may be located on P1 and P2. In general, key functional regions of biomolecules (e.g., binding sites) are more sensitive to residue mutations.^44^ We further mutated C24, G27, C28, G34, G38, C39, C40, G42, and C44 to A24, T27, A28, T34, T38, A39, A40, T42, and A44 (Fig. S7). The binding of the aptamer to the SARS-CoV-2 N protein was determined via ELONA (Fig. 3G). The nucleotides in region P1 (namely C24 and G27) may be important functional components of NP14, and at the same time, P2 is also a part of the nucleotides that maintain the secondary structure of NP14 and cannot be removed. Previous studies have suggested that some aptamers or guanine-rich sequences may form G-quadruplex structures, which can impact their binding properties.^45^ To explore whether NP14 adopts this structure, we analyzed its circular dichroism (CD) spectrum. Typical G-quadruplexes show positive peaks at 260 or 290 nm and negative peaks near 240 nm.^45^ AS1411, which has multiple G-quadruplex conformations, was used as a control.^46^ As shown in Figure 3H, NP14 exhibited a positive peak at 280 nm and a negative peak at 250 nm, which differ from the canonical G-quadruplex spectra. This result indicates that NP14 does not form a G-quadruplex but is more likely to adopt a B-form or hairpin-like DNA structure.^43^ Because G-quadruplexes often interact promiscuously with proteins owing to their conserved topology and electrostatic properties,^47^ the deviation of NP14 from this structure may contribute to its enhanced selectivity. The SARS-CoV-2 N protein is composed primarily of the NTD and C-terminal domain (CTD), as shown in Figure 3I. To further pinpoint the binding site of NP14, we expressed the amino acid domain containing the NTD segment in a eukaryotic system (Fig. S8), and the recognition of the Flag-SARS-CoV-2 N1-ZS Green protein by NP14 was subsequently validated via immunomagnetic bead-based chemistry technology (Fig. S9). As shown in Figure 3J, NP14 specifically recognized the amino acid sequence containing the NTD segment compared with the blank control group. According to prior studies, aptamers A58 and A61 bind to the NTD, whereas N1 does not.^18^ Information on N1, A58, and A61 is provided in Table S3. A competitive binding assay was conducted using biotin-labeled NP14 and unlabeled aptamers targeting NTD or non-NTD regions. We observed that N1 does not compete with NP14, whereas A58 and A61 compete with NP14 (Fig. 3K), indicating that NP14 binds the NTD, which is consistent with our docking and domain-targeting data. Moreover, NP14 exhibited a binding affinity of 7.64 nM for the NTD-containing protein, indicating better NTD-specific binding among currently known aptamers (Fig. 3L). This finding elucidates the new binding mechanism of NP14 to the SARS-CoV-2 N protein. To assess cross-reactivity, we further evaluated the effects of NP14 truncation and mutation on its binding to the SARS-CoV N protein using ELONA. As shown in Figure S10B, the truncated variants NP14a and NP14b had minimal effects on binding, whereas base-mutated variants (NP14a1, NP14a2, NP14b1, and NP14b2) significantly reduced binding activity. This finding suggests that the P1 region nucleotides, especially C24 and G27, are also critical for recognition of the SARS-CoV N protein. The limited impact of truncation may be related to structural differences in the N protein binding site.^48^ In summary, we speculate that NP14 binds to a conserved epitope within the N-terminal domain of the SARS-CoV-2 N protein through key interactions involving nucleotides C24 and G27 of the P1 region, whereas the P2 region is essential for maintaining the secondary structure of NP14. These findings provide structural insight into aptamer–protein recognition and lay the groundwork for rational optimization of aptamer-based diagnostics.

### Specificity and cross-variant recognition of NP14

**Figure 4:**
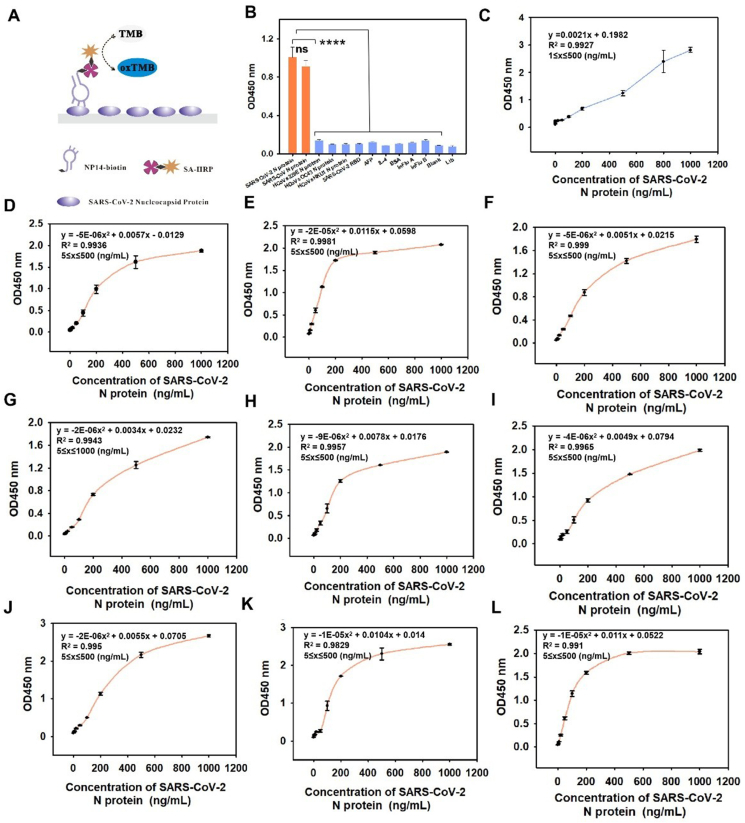
Specificity and cross-variant recognition of NP14 for the SARS-CoV-2 N protein. **(A)** ELONA method detection mode diagram. **(B)** NP14 labeled with 400 nM biotin was used with various proteins (1 μg/mL): SARS-CoV N protein, human coronavirus (HCoV) 229E, OC43, HKU1, SARS-CoV-2 receptor-binding domain (RBD), alpha-fetoprotein (AFP), interleukin-4 (IL-4), bovine serum albumin (BSA), and influenza (InFlu) A and B proteins, to validate the specificity of NP14 via ELISA. Data were presented as mean ± standard deviation of triplicate results (*n* = 3). Compared with the SARS-CoV-2 N protein: ns, not significant; ∗∗∗∗*p* < 0.0001. **(C)** Direct detection of SARS-CoV-2 N protein binding activity at various concentrations (0, 0.5, 1, 5, 10, 20, 50, 100, 200, 500, 800, and 1000 ng/mL) via the ELONA platform. Data were presented as mean ± standard deviation of triplicate results (*n* = 3). **(D**–**L)** Detection of NP14 (biotin-labeled, 400 nM) binding to N recombinant proteins from SARS-CoV-2 variants at different concentrations (0, 5, 10, 20, 50, 100, 200, 500, and 1000 ng/mL) on the direct ELONA platform. Variants included (D) alpha, (E) beta, (F) gamma, (G) delta, (H) omicron B.1.640, (I) omicron BA.2, (J) lambda, (K) omicron BA.1, and (L) omicron BA.4.

To evaluate the specificity of NP14 and its ability to detect the SARS-CoV-2 N protein across multiple viral variants, we conducted a series of binding assays. Building on prior findings that NP14 targets a partially conserved region within the N protein, we sought to assess both its selectivity and variant coverage. We first performed an ELONA assay using 400 nM NP14 to characterize its binding profile against a diverse panel of viral and nonviral proteins (Fig. 4A). NP14 was tested against a panel of viral and control proteins, including SARS-CoV N protein, human coronavirus (HCoV) 229E, OC43, HKU1, the SARS-CoV-2 receptor-binding domain (RBD), alpha-fetoprotein (AFP), interleukin-4 (IL-4), BSA, influenza (InFlu) A and B proteins, and the SARS-CoV-2 N protein. A blank PBS buffer (0.01 M, pH 7.4) was included as a negative control. Proteins such as BSA, IL-4, and AFP were selected as standard negative controls commonly used in biochemical assays, while viral proteins (e.g., InFlu A/B and the RBD) served as related but non-target comparisons. Notably, NP14 exhibited high specificity, as it strongly bound to the SARS-CoV-2 N protein without significant cross-reactivity with other viral or control proteins (Fig. 4B). Although the N protein is relatively conserved, the ability to recognize different variants is crucial for broad-spectrum detection. To further evaluate this, we assessed the binding activity of NP14 (400 nM) against multiple SARS-CoV-2 variants using ELONA. By titrating various concentrations of the original recombinant SARS-CoV-2 N protein (Fig. 4C), alpha (Fig. 4D), beta (Fig. 4E), gamma (Fig. 4F), delta (Fig. 4G), omicron B.1.640 (Fig. 4H), omicron BA.2 (Fig. 4I), lambda (Fig. 4J), omicron BA.1 (Fig. 4K), and omicron BA.4 (Fig. 4L), we determined that for the original recombinant SARS-CoV-2 N protein, the linear regression equation can be expressed as *y* = 0.0021*x* + 0.1982 (*R*^*2*^ = 0.9927), with a dynamic detection range of 1–500 ng/mL (Fig. 4C). NP14 maintained detectable binding activity toward all the tested variants even at a low concentration of 5 ng/mL. The experimental results show that NP14 has a high binding capacity for various species of the SARS-CoV-2 N protein. Compared with that of the prototype, the binding of the aptamer to the mutant strain was slightly reduced, likely due to spatial structural changes in the N protein of the variants.^48^ Notably, NP14 consistently bound to alpha, beta, gamma, delta, omicron (BA.1, BA.2, B.1.640, BA.4), and lambda variants across a wide concentration range, confirming its broad-spectrum diagnostic potential (Fig. 4D–L). This broad-spectrum recognition underscores the utility of NP14 for variant-resilient antigen detection platforms.

In conclusion, NP14 demonstrates high specificity and broad-spectrum variant recognition, supporting its utility in mutation-resilient diagnostics.

### Diagnostic comparison of the performance of aptamer-antibody and double antibody detection

**Figure 5:**
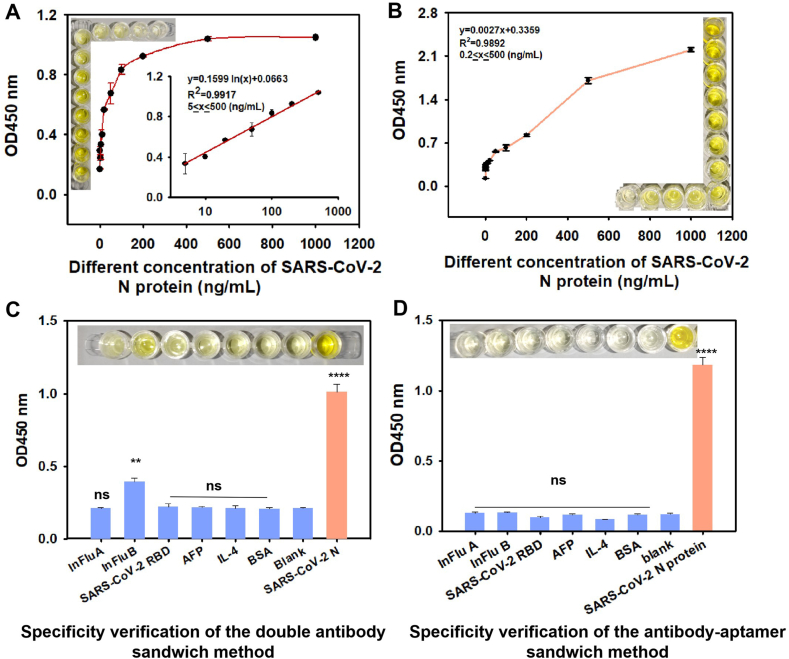
Comparative sensitivity and specificity of antibody–antibody versus antibody–aptamer sandwich assays. **(A)** Standard curve for the sandwich assay (1 μg/mL antibody) using the SARS-CoV-2 N protein at various concentrations (0, 0.1, 0.5, 1, 5, 10, 20, 50, 100, 200, 500, and 1000 ng/mL). Data were presented as mean ± standard deviation of triplicate results (*n* = 3). **(B)** Standard curve of the SARS-CoV-2 N protein in the antibody‒aptamer sandwich mode using SARS-CoV-2 N protein at various concentrations (0, 0.2, 0.5, 1, 5, 10, 20, 50, 100, 200, 500, and 1000 ng/mL). Data were presented as mean ± standard deviation of triplicate results (*n* = 3). **(C)** Specificity validation with multiple proteins (1 μg/mL), including: SARS-CoV-2 receptor-binding domain (RBD), alpha-fetoprotein (AFP), interleukin-4 (IL-4), bovine serum albumin (BSA), influenza (InFlu) A and B proteins, to validate the specificity of the antibody–antibody (1 μg/mL) sandwich assay. Data were presented as mean ± standard deviation of triplicate results (*n* = 3). Compared with the blank control: ns, not significant; ∗∗*p* < 0.01 and ∗∗∗∗*p* < 0.0001. **(D)** Validation was performed using multiple proteins at a concentration of 1 μg/mL, including: SARS-CoV-2 RBD, AFP, IL-4, BSA, InFlu A and B proteins, to validate the specificity of the antibody (1 μg/mL)-aptamer (200 nM) sandwich assay. Data were presented as mean ± standard deviation of triplicate results (*n* = 3). Compared with the blank control: ns, not significant; ∗∗∗∗*p* < 0.0001.

To evaluate the diagnostic potential of the NP14 aptamer, we developed an ELAAA that targets the SARS-CoV-2 N protein. Previous studies have demonstrated that antibody–aptamer sandwich assays offer higher sensitivity and selectivity than conventional double-antibody sandwich methods do, thereby addressing several limitations of the latter.^12^^,^^49^ These advantages are often attributed to the reduced steric hindrance and improved molecular accessibility afforded by the smaller size and structural flexibility of the aptamers. To validate this concept in our platform, we directly compared the performance of the NP14-based aptamer–antibody hybrid assay with that of a traditional double-antibody sandwich assay. The double-antibody sandwich assay demonstrated a linear detection range of 5–500 ng/mL with a limit of detection (LOD) of 0.5 ng/mL (Fig. 5A). In contrast, the aptamer-antibody sandwich assay showed superior sensitivity, achieving an LOD of 0.1 ng/mL with an extended dynamic range of 0.2–500 ng/mL (Fig. 5B). This enhanced performance suggests that the aptamer-antibody combination provides more accessible binding sites while maintaining high target specificity. Moreover, to verify the specificity of the double-antibody and aptamer-antibody sandwich modules, solutions containing 500 ng/mL SARS-CoV-2 N protein in negative PBS buffer (0.01 M, pH 7.4) were prepared alongside solutions of the SARS-CoV-2 RBD, AFP, IL-4, BSA, InFlu A, and InFlu B proteins. Additionally, a blank control (negative PBS) was included. As shown in Figure 5C, the results of specificity testing using a double-antibody sandwich ELISA indicated that, compared with the blank control, the assay had a highly significant ability to detect the SARS-CoV-2 N protein (*p* < 0.0001). However, InFlu B also produced a marginally significant signal (*p* < 0.01), indicating partial cross-reactivity and thus reduced specificity for the SARS-CoV-2 N protein. In contrast, Figure 5D shows that specificity testing with the antibody–aptamer sandwich method revealed no significant differences for other non-target proteins, whereas the targeted SARS-CoV-2 N protein exhibited a highly significant signal (*p* < 0.0001). The results show that the aptamer-antibody sandwich method results in low nonspecific adsorption. All the experiments were repeated three times. In summary, the detection of SARS-CoV-2 N protein via the antibody–aptamer hybrid assay yielded a lower background, higher sensitivity, and broader dynamic range than did the conventional antibody–antibody sandwich method. These improvements may result from the flexible secondary structure of the aptamers, which enables enhanced binding in concert with the antibodies.^11^ Overall, the antibody–aptamer hybrid format significantly enhances diagnostic performance in SARS-CoV-2 antigen detection.

### Design and validation of the dual-mode MD ELAAA detection platform

**Figure 6:**
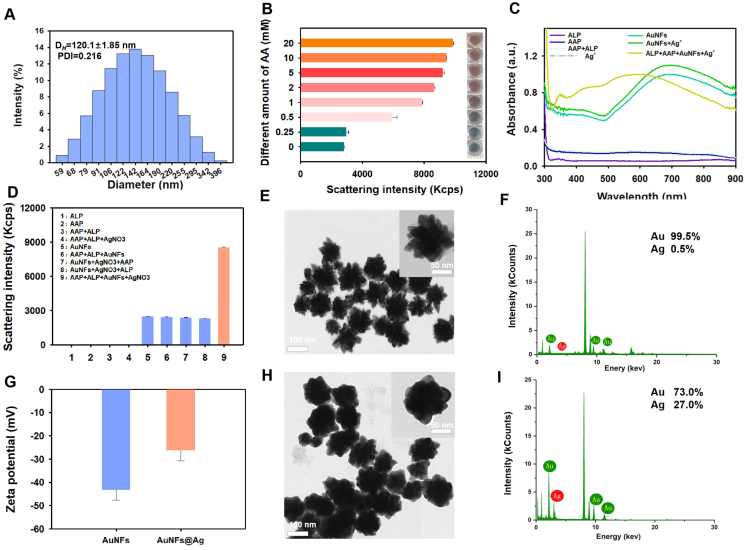
Validation of the dual-mode detection platform combining multicolor visualization and dynamic light scattering (DLS). **(A)** Size distribution of the AuNFs (5 pM) by DLS. **(B)** Proof-of-concept dual-mode detection of silver deposition on AuNF (100 pM) surfaces via reduction of AgNO_3_ (10 μL, 0.01 M) at different AA concentrations (0, 0.25, 0.5, 1, 2, 5, 10, and 20 mM). **(C)** UV–vis spectra of ALP, AAP, ALP + AAP, AgNO_3_, AuNFs, AuNFs + AgNO_3_, and ALP + AAP + AuNFs + AgNO_3_. ALP: Tris–HCl diluted 300-fold; AAP: 6 mM; AuNFs: 100 pM; AgNO_3_: 0.01 M). **(D)** Scattering intensities of ALP, AAP, ALP + AAP, AAP + ALP + AgNO_3_, AuNFs, AAP + ALP + AuNFs, AuNFs + AgNO_3_ + AAP, AuNFs + AgNO_3_ + ALP, and ALP + AAP + AuNFs + AgNO_3_. ALP: Tris–HCl diluted 300-fold; AAP: 6 mM; AuNFs: 100 pM; AgNO_3_: 0.01 M. **(E)** Transmission electron microscopy image of the AuNFs. At 100 nm and 50 nm fields of view, AuNFs exhibited a spike-like morphology. **(F)** Energy dispersive spectrometer (EDS) of the AuNFs. Au content: 99.5%; Ag content: 0.5%. **(G)** Zeta potential (mV) of the AuNFs and AuNFs@Ag in ddH_2_O_2_ buffer. Data were presented as mean ± standard deviation of triplicate results (*n* = 3). **(H)** Transmission electron microscopy image of the AuNFs@Ag at 100 nm and 50 nm fields of view. **(I)** EDS spectrum of AuNFs@Ag. Au content: 73.0%; Ag content: 27.0%.

To integrate NP14 into a dual-mode detection platform combining colorimetry and DLS, we extended the aptamer-antibody sandwich format to develop an MD ELAAA. This approach was designed to improve detection performance through simultaneous visual and quantitative readouts. As illustrated in Figure 1B, the platform detects the SARS-CoV-2 N protein based on DNA aptamer recognition, utilizing both plasmonic color changes and light scattering intensity as output signals. In the assay, a capture antibody was first immobilized on a 96-well microtiter plate, followed by binding of the SARS-CoV-2 N protein from the test sample and subsequent hybridization with biotin-labeled aptamer NP14. A lower concentration of the target protein resulted in reduced SA-ALP binding. ALP then catalyzed the hydrolysis of AAP to ascorbic acid (AA). In the colorimetric vessel of the assay, AA reduced Ag^+^ to silver monomers, which were deposited *in situ* onto AuNFs to form silver-coated AuNF@Ag nanostructures.^50^ As the silver shell thickened, the AuNF@Ag structures underwent progressive blueshifts in their localized surface plasmon resonance (LSPR), producing noticeable color changes. This enabled multicolor detection of the SARS-CoV-2 N protein for qualitative or semiquantitative visual analysis. For the DLS assay, the scattering intensity of the AuNF@Ag nanocomposites demonstrated a good linear relationship with the AA concentration, enabling precise quantification of protein detection. Since 120 nm AuNFs possess an excellent molar extinction coefficient as well as colloidal stability,^51^ we synthesized chestnut shell-like gold nanomaterials with an average particle size of 120.1 ± 1.85 nm by seeded gold growth development. The UV and hydrated particle sizes of the seed gold are shown in Figure S11. The successful synthesis of the AuNFs was verified via DLS (Fig. 6A). To confirm the feasibility of AA-induced silver deposition on the AuNFs, several controlled experiments were conducted. The scattering intensity values of the AuNF@Ag nanocomposites gradually increased with increasing AA concentration, accompanied by significant color changes (Fig. 6B). Additionally, as demonstrated in Figure 6C, compared with the original AuNFs, the resultant AuNFs, AAP, alkaline phosphatase (ALP), and AgNO_3_ exhibited evident blueshifts in the UV–vis absorption spectra from 688 nm to 588 nm. However, the UV–vis absorption spectra of AAP, Ag^+^, and ALP solutions did not exhibit significant absorbance peaks in the visible region from 300 nm to 900 nm. Similarly, as shown in Fig. 6D, the increase in the scattering intensity of the synthesized AuNFs, AAP, ALP, and AgNO_3_ was greater than that of the pristine AuNFs. However, the AAP, Ag^+^, and ALP solutions did not exhibit significant scattering intensity. The prepared AuNFs with Ag-coated AuNF nanocomposites (AuNF@Ag) were characterized via transmission electron microscopy. Transmission electron microscopy images (Fig. 6E and H) show that when AA is generated in the presence of AAP and ALP, and then under lower concentrations of AgNO_3_, the silver coating is relatively thin, and the branched morphology of the AuNFs remains partially visible, resulting in irregular AuNFs@Ag. Additionally, energy dispersive X-ray elemental mapping images of the AuNFs and the spectral deposition of AuNFs@Ag were obtained before further analysis (Fig. 6E and F); and after analysis (Fig. 6H and I), the deposition reaction results were obtained, further confirming that AA induced the deposition of silver on the AuNFs. Furthermore, the zeta potential significantly increased from −43 to −26 mV for the AuNFs@Ag compared with the original AuNFs (Fig. 6G). These results confirm that the ALP–AA–Ag^+^ reaction system effectively drives silver deposition on AuNFs, triggering LSPR and scattering changes. The MD ELAAA platform, therefore, enables both naked-eye qualitative detection and DLS-based quantitative measurement, expanding the diagnostic capabilities of aptamer-based biosensing.

### Analytical performance evaluation of the MD ELAAA method

**Figure 7:**
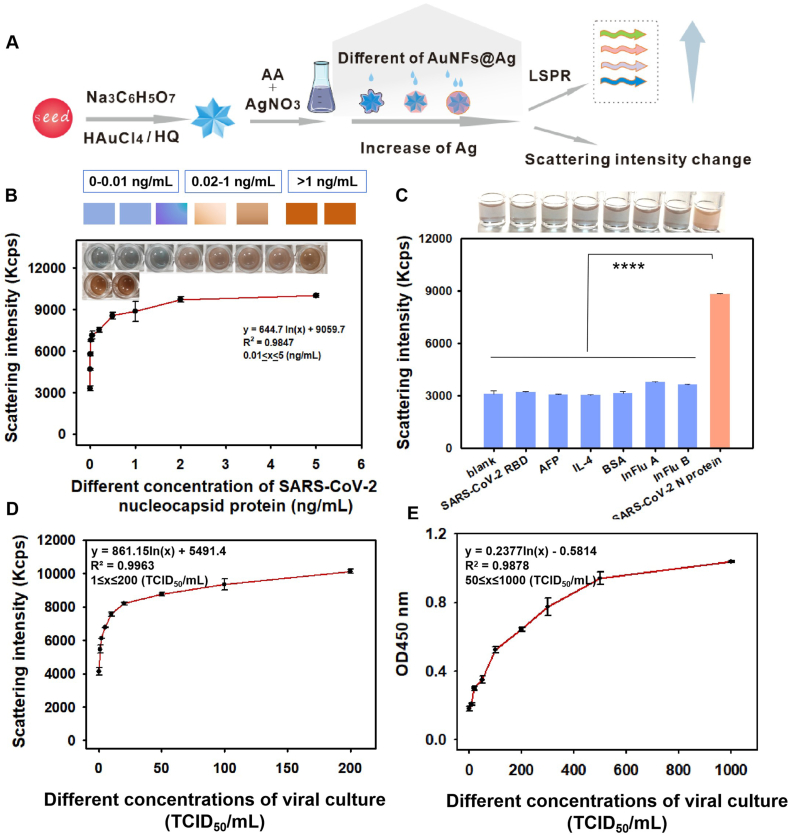
Analytical performance of the MD ELAAA platform in detecting the SARS-CoV-2 N protein and viral cultures. **(A)** Schematic illustration of the modulation of the Ag shell layer thickness in core–shell AuNFs@Ag nanostructures leading to changes in the localized surface plasmon resonance (LSPR) and light scattering intensity. **(B)** Standard curve of the MD ELAAA method for different SARS-CoV-2 N proteins (0, 0.005, 0.01, 0.02, 0.05, 0.1, 0.5, 1, 2, and 5 ng/mL). Data were presented as mean ± standard deviation of triplicate results (*n* = 3). **(C)** Validation was performed using multiple proteins at a concentration of 1 ng/mL, including: SARS-CoV-2 receptor-binding domain (RBD), alpha-fetoprotein (AFP), interleukin-4 (IL-4), bovine serum albumin (BSA), influenza (InFlu) A and B proteins, to validate the specificity of the MD ELAAA platform. Data were presented as mean ± standard deviation of triplicate results (*n* = 3). The blank control: ns, not significant; ∗∗∗∗*p* < 0.0001. **(D)** Standard curve of the MD ELAAA method for SARS-CoV-2 virus cultures at different concentrations (0, 1, 2, 5, 10, 20, 50, 100, and 200 TCID_50_/mL). Data were presented as mean ± standard deviation of triplicate results (*n* = 3). **(E)** Standard curve of the ELAAA method for SARS-CoV-2 virus cultures at different concentrations (0, 10, 20, 50, 100, 200, 300, 500, and 1000 TCID_50_/mL). Data were presented as mean ± standard deviation of triplicate results (*n* = 3).

To assess the practical diagnostic performance of the MD ELAAA platform, we conducted a series of experiments using both recombinant proteins and virus cultures. Before these evaluations, we optimized several key assay parameters to ensure maximal sensitivity and reproducibility. In the multicolor analysis, signal generation depends on the silver deposition reaction driven by AA, which itself is produced by the ALP-catalyzed hydrolysis of AAP. To fine-tune this reaction, we systematically optimized the concentrations of AAP, silver nitrate (AgNO_3_), and AuNFs, as well as the pH of the Tris–HCl buffer.^50^ As shown in Figure S12, the optimal experimental conditions were as follows: 6 mM for AAP, Tris–HCl (0.05 M, pH 8.8), 180 nmol for AgNO_3_, and 10.8 nmol for the AuNFs. The SARS-CoV-2 N protein was detected via a dual-mode aptasensor with multicolor and light dispersive intensity under optimized conditions. Dual-mode signal analysis was conducted by observing the changes in color of the SPR and the light scattering intensity resulting from silver deposition on the surface of the AuNFs, which was mediated by the specific reaction of the aptamer with the protein (Fig. 7A). As shown in Figure 7B, as the concentration of the SARS-CoV-2 N protein increased from 0 to 5 ng/mL, a clear color change was observed, with the color changing from blue to light red to brownish red. When the concentration of the SARS-CoV-2 N protein was less than 0.02 ng/mL, the solution remained blue. However, when the concentration of the SARS-CoV-2 N protein exceeded 0.02 ng/mL, the color gradually shifted from blue to light red and then to brownish red. Therefore, this multicolor change can be easily recognized by the naked eye, facilitating device-free semiquantitative detection of the SARS-CoV-2 N protein. Through DLS quantitative detection, the linear regression equation can be expressed as y = 644.7ln(x) + 9059.7 (R^2^ = 0.9847) with a dynamic detection range of 0.01–5 ng/mL. Furthermore, the limit of detection (LOD) of the MD ELAAA method was 0.39 pg/mL, which was calculated via the following formula: LOD = y blank + 3 × SD blank, where y blank is the scattering intensity of the blank control, and SD blank is the corresponding standard deviation.^52^ To verify the specificity of the dual-mode aptasensor, solutions of the SARS-CoV-2 RBD, AFP, IL-4, BSA, InFlu A proteins, InFlu B proteins, and SARS-CoV-2 N protein were tested in negative lysis buffer along with a blank control (negative lysate). The responses induced by these proteins were comparable to those induced by the blank, indicating that the aptasensor exhibited good selectivity for the SARS-CoV-2 N protein (Fig. 7C). In addition, we evaluated a commercial ELISA kit with a sensitivity of 1.4 ng/mL (Fig. S13). Our methodology demonstrated superior detection performance for the SARS-CoV-2 N protein compared with commercial ELISA kits. Additionally, a standard curve for SARS-CoV-2 variants, including alpha, beta, gamma, delta, omicron BA.1, and Lambda, was successfully established using the MD ELAAA assay (Fig. S14). These findings highlight the platform's diagnostic potential for early SARS-CoV-2 detection and variant surveillance.

To further evaluate the effectiveness of the developed aptamer-mediated dual-mode sensor method for the detection of authentic viruses, we examined SARS-CoV-2 virus cultures. We subsequently utilized negative lysates spiked with SARS-CoV-2 virus cultures for separate assays via the ELAAA and MD ELAAA methods. The cutoff value demonstrated in Figure 7D was 0.43 TCID_50_/mL for inactivated viral cultures, defined as the mean value determined in the absence of antigen plus three times the standard deviation. Compared with that of ELAAA, the sensitivity of the enhanced dual readout strategies improved by 47-fold (Fig. 7E). Additionally, we compared our assay with other published aptamer-based detection methods, as summarized in Table S4. The MD ELAAA system showed superior sensitivity, outperforming most previously reported platforms.

The MD ELAAA platform exhibits exceptional sensitivity, specificity, and practicality for detecting both the recombinant SARS-CoV-2 N protein and viral cultures in lysates. These results highlight its strong potential for clinical and point-of-care diagnostic applications.

## Discussion

The development of highly sensitive, high-throughput, and effective diagnostics for pathogenic coronaviruses will be important in establishing methodologies for the ongoing SARS-CoV-2 pandemic. This study substantially advances SARS-CoV-2 diagnostics by targeting the critical N protein, which plays a key role in viral RNA binding, packaging, and assembly. First, we obtained the target SARS-CoV-2 N protein DNA aptamer NP14 via a computer-assisted X-Aptamer SELEX strategy. Next, the binding mechanism of NP14 to the SARS-CoV-2 N protein was explored in terms of different aspects. We investigated NP14 binding by truncating and mutating the aptamer itself, as well as by truncating the SARS-CoV-2 N protein. Furthermore, the MD ELAAA method was constructed to detect the SARS-CoV-2 N protein with multiple detection capabilities. Importantly, SPR and ELONA serve complementary roles in assay validation. SPR provides quantitative kinetic parameters under controlled buffer conditions, whereas ELONA mimics solid-phase diagnostic environments, offering predictive value for real-world applications. Together, these methods ensure both the biophysical validity and diagnostic robustness of NP14 performance.

Conventional protein SELEX often requires multiple repetitions, which are inefficient and time-consuming.^53^^,^^54^ However, our approach reduces screening time, eliminates the need for antibodies, and minimizes protein usage, thereby streamlining the aptamer development process. Here, our screen identified an efficient and selective DNA aptamer, NP14, that binds to all tested SARS-CoV-2 variants, as well as to the N protein of SARS-CoV, and does not bind to other coronavirus N proteins. Because the SARS-CoV-2 N protein shares 91 % homology with the SARS-CoV N protein,^55^ NP14 also binds well to the SARS-CoV N protein. Similarly, Chen et al reported that some early DNA aptamer N1 targeting SARS-CoV N proteins were able to bind to SARS-CoV-2 N proteins with a K_D_ of 4.93 ± 0.30 nM and a length of 88 nt, but NP14 had a superior K_D_ of 3.45 nM (Fig. S10A) with a shorter sequence.^56^^,^^57^ These findings highlight the successful generation of new high-affinity SARS-CoV N and SARS-CoV-2 N protein aptamers as valuable tools for the detection of the SARS-CoV N and SARS-CoV-2 N proteins in diagnostic applications.

Our findings broaden the understanding of the mechanism of action of DNA aptamers on the SARS-CoV-2 N protein. The binding between aptamers and proteins is mediated by various molecular interactions such as hydrogen bonds, electrostatic attractions, hydrophobic contacts, and van der Waals forces.^19^^,^^58^ Notably, the principles and mechanisms involved in target recognition are vital to the development of more sensitive detection methods. Recently, studies have revealed the binding mechanism of aptamer A58-20, which is centered on a hexanucleotide loop (5′-TCGGAT-3′) that binds the SARS-CoV-2 NTD structural domain.^18^ However, the structure and base sequences of different aptamers also affect their binding activity to the SARS-CoV-2 N protein.^19^ Our experimental results show that NP14 binds to a conserved region in amino acids 1–219 (Fig. 3J and K), providing an important structural basis for the detection and treatment of different SARS-CoV-2 variants using aptamers. On the other hand, we have systematically explored the binding mechanism in terms of proper truncation and mutation of the aptamers. For example, He et al used sgc8c as an object of study and identified the key site of PTK7 binding by sgc8c by means of NMR, stepwise truncation, and targeted mutagenesis.^44^ We investigated the key binding regions of NP14 through a combination of molecular docking and ELONA-based wet-lab experiments, providing deeper insights into the interaction between NP14 and the SARS-CoV-2 N protein. As shown in Figure 3A–G, key nucleotides in the P1 region, particularly C24 and G27, were presumed to be critical binding sites, whereas the P2 region is essential for maintaining the secondary structure of NP14. Notably, the dissociation constant of our aptamer NP14 for NTD (K_D_ = 7.64 nM) was greater than that of the A58-20 aptamer (K_D_ = 101 nM)^18^ which has been reported, which may be attributed to the fact that the key binding site, G-C, in NP14 enhances the affinity for strengthening the stem region, which is in agreement with previous reports.^59^ Research has demonstrated that the N-terminal domain of the NTD tends to bind to stem-loop structures within the viral genome.^60^^,^^61^ The observed recognition of the P1 and P2 regions by the SARS-CoV-2 N protein may reflect a mechanism that mirrors its interaction with RNA in a biologically relevant context. These studies suggest a binding mechanism between NP14 and the SARS-CoV-2 N protein. Although the role of NP14 with the SARS-CoV-2 N protein needs to be proven by further characterization in terms of its crystal structure, revealing the binding between NP14 and the SARS-CoV-2 N protein will prompt us to further investigate its potential for molecular detection and therapy, and will help to understand the recognition ability of NP14 in detection from another perspective. Above all, our research offers a new structural understanding of how a DNA aptamer binds to the SARS-CoV-2 N protein, identifying the specific binding site that is crucial for optimizing the aptamer for diagnostic use.

Currently, the role of DNA aptamers in SARS-CoV-2 antigen detection has been demonstrated in early studies (Table S4), including nLC-MS/MS and XPS analysis; however, these methods demonstrate strong quantitative accuracy and they face significant limitations, including prolonged detection times, the inability to process multiple samples in parallel, reliance on expensive equipment and specialized expertise, complex sample preparation protocols, and a lack of portability for on-site analysis.^62^ The emergence of electrochemical biosensors has provided alternative options, although they are still constrained by stability issues, such as susceptibility to electrode surface contamination, which can lead to performance degradation.^63^ Additionally, aptamer-based immunofluorescence sensing technology has emerged,^64^ but it requires instrumentation to obtain signal output. To address this issue, immunochemical techniques offer advantages such as high specificity, high sensitivity, ease of operation, rapid detection, and compatibility with automation. Among them, Immunochemical methods for antibody and aptamer sandwich patterns offer advantages such as the ability to simultaneously detect multiple samples and rapid analysis.^16^ Currently, the antibody and aptamer sandwich model is used for the detection of viruses,^16^^,^^64^, ^65^, ^66^ mycotoxins,^11^^,^^67^ molecule biotoxins,^68^ growth factor,^69^ thrombin,^49^^,^^70^ and cancer.^71^ It has a higher positive predictive value and negative predictive value, and lower false positive and false negative rates than the traditional double antibody sandwich method does,^11^^,^^49^^,^^71^ similar to the results we reported (Fig. 5). These data and reports suggest that the antibody-aptamer sandwich model may be valuable.

Sensitivity and specificity are important metrics for detection methods, so we developed an innovative MD ELAAA detection platform that broadens the application platform for the antibody-aptamer sandwich model (Fig. 1B). The MD ELAAA leverages dual-mode non-aggregation plasmonic colorimetry and light scattering intensity from Au/Ag nanomaterials, enabling semi-quantitative visual detection and precise instrumental quantification. This approach achieves a detection limit of 0.43 TCID_50_/mL, surpassing the sensitivity of traditional assays by 47-fold (Fig. 7C). The detection capabilities of our developed MD ELAAA method outperform those of most previously reported techniques (Table S4). The MD ELAAA method has higher sensitivity for several reasons. On one hand, the newly identified recognition element NP14 has high affinity and excellent specificity, and the determination of its specific binding site is crucial for developing more sensitive detection methods. On the other hand, by leveraging the unique advantages of antibody specificity and aptamer affinity, we combined the anti-SARS-CoV-2 N protein antibody with the aptamer, thereby increasing detection sensitivity. Most importantly, we integrated light scattering intensity with multicolor ELAAA, which not only enables naked-eye detection but also allows for highly sensitive quantitative detection. Among all nanobiosensors, plasmonic nanosensors that utilize the SPR properties of AuNFs are of great interest. By controlling the structure of the AuNFs, the LSPR absorption and color of the solution can be adjusted, providing an excellent platform for the development of highly sensitive biochemical analyses that can be read easily.^72^^,^^73^ DLS is a powerful analytical method used to determine both the intensity of scattered light and the mean hydrodynamic diameter (D_H_) of particles in suspension. According to theoretical principles, the light scattering signal from AuNFs is more sensitive than their absorption signal is, as the scattering intensity scales as the particle radius increases to the sixth power.^40^ Therefore, this is also one of the reasons why the MD ELAAA method exhibits high sensitivity. In brief, we have expanded the antibody-aptamer sandwich mode to a dual-mode platform of multicolor and DLS, leveraging its versatility and high sensitivity for biochemical detection in multi-scenario applications. These innovations represent significant advancements in diagnostic technologies, providing versatile capabilities that are critical for effective disease management in clinics.

Nevertheless, the study acknowledges several limitations. The current workflow involves multiple steps, which may hinder its application in point-of-care settings. Therefore, the process could be streamlined, for example, by directly conjugating the aptamer with peroxidase and integrating it with portable devices for on-site diagnostic deployment. Future work will focus on optimizing detection conditions to meet true point-of-care requirements and expanding detection capabilities to include SARS-CoV-2 S proteins. In addition to multicolor detection, the MD ELAAA system also supports target discrimination using distinct light scattering signatures, which can be visually assessed or captured through smartphone-assisted platforms-eliminating reliance on microplate readers. Notably, studies by Luo et al^50^ and Guo et al^74^ have demonstrated the feasibility of coupling colorimetric or fluorescence-based outputs with smartphone cameras and custom applications, enabling rapid and quantitative point-of-care testing even in resource-limited environments. Moreover, the application of machine learning to analyze multicolor and scattering signals can transform simple optical cues into digital outputs. Zhou et al. developed the first machine learning model targeting the odor characteristics of odor-causing substances in water, achieving accurate prediction of odor categories and odor thresholds of odor-causing substances based on molecular structure or secondary mass spectrometry data.^75^ Yu et al developed a rapid, simple, and sensitive technique to determine the antibiotic resistance phenotype of ESKAPE pathogens by combining plasma nanoscale sensors and machine learning. This machine learning-based plasma method has the advantages of being easy to operate and relatively low in cost.^73^ The artificial intelligence model based on computer vision constructed by Zhao et al can analyze microsphere images to decode information and output the identification results in a visual manner.^76^ Guo et al developed a hybrid chain reaction system based on a rectangular DNA framework and constructed a dual-mode EV-circRNA *in situ* analyzer (BEISA).^77^ The analyzer can provide dual signal output in both fluorescence and electrochemical modes. Combining BEISA with machine learning technology provides an efficient and reliable tool for EV-circRNA analysis, which is helpful for the early diagnosis of gastric cancer.^77^ Based on these studies, MD ELAAA has the potential to provide a multifunctional diagnostic approach that does not require instruments by using a mobile application to convert color codes and scattering patterns into biomarker concentrations. Given the high throughput, high sensitivity, and good specificity of MD ELAAA, the improved digital MD ELAAA method is expected to become a smart analytical method for next-generation multifunctional biosensing. This combination of multicolor scattering signals and AI-driven interpretation supports portable, user-friendly diagnostic kits ideal for point-of-care use in rural clinics, mobile health units, and resource-limited areas. Furthermore, the therapeutic potential of SARS-CoV-2 N protein aptamers, particularly their role in disrupting viral genome assembly and immune evasion, merits further investigation.

In summary, we developed NP14, a high-affinity DNA aptamer that targets a conserved region in the N-terminal domain of the SARS-CoV-2 N protein, using X-aptamer SELEX and computational docking. NP14 showed spectral binding across multiple variants, with key nucleotides C24 and G27 identified as critical for interaction. Leveraging this, we established a dual-mode biosensing platform (MD ELAAA) that integrates plasmonic colorimetry and dynamic light scattering for both visual and quantitative detection. The system achieved a detection limit of 0.43 TCID_50_/mL, a 47-fold improvement over standard ELISA. These results advance the structural understanding of aptamer–N protein recognition and highlight the diagnostic potential of this platform for early-stage SARS-CoV-2 detection and variant monitoring.

## CRediT authorship contribution statement

**Shu Zhou:** Writing – original draft, Validation, Investigation, Formal analysis. **Yuxi Xu:** Visualization, Software, Formal analysis. **Huan Liao:** Formal analysis, Investigation. **Hailong Ou:** Formal analysis, Data curation. **Dan Qi:** Writing – review & editing, Supervision, Formal analysis. **Yatao Wu:** Writing – review & editing, Investigation, Data curation. **Yunyi Liu:** Methodology, Data curation. **Juan Li:** Investigation. **Jiaxuan Li:** Formal analysis. **Bi Shi:** Resources. **Fei Zhu:** Formal analysis. **Siran Zhang:** Investigation. **Jason H. Huang:** Writing – review & editing, Resources. **Erxi Wu:** Writing – review & editing, Supervision, Resources, Funding acquisition. **Xiaoxiao Hu:** Supervision, Resources, Funding acquisition, Conceptualization.

## Data availability

The data will be made available upon request.

## Funding

This study was supported by the Natural Science Foundation of Hunan, China (No. 2023JJ30124 to X. Hu; 2025JJ60169 to Y. Liu), the Natural Science Foundation of Chongqing, China (No. CSTB2022NSCQ-MSX1551 to X. Hu), the Natural Science Foundation of Fujian, China (No. 2023J01247 to X. Hu), the Natural Science Foundation of Guangdong, China (No. 2024A1515012771 to X. Hu), the Postdoctoral Fellowship Program of Chinese Postdoctoral Science Foundation (CPSF) (No. GZB20240215 to Y. Liu), the Hunan Science and Technology Innovation Plan (No. 2025ZYJ003), and the Corbett Estate Fund for Cancer Research (No. 62285-531021-41800, 62285-531021-51800, 62285-531021-61800, and 62285-531021-71800 to E. Wu).

## Conflict of interests

Erxi Wu is one of the Associate editors of *Genes & Diseases*, but has no invlovement in the peer review of this manuscript. The authors have no other competing interests to declare.
