# Supplementary material for: Dual-mode aptamer-driven biosensing platform for ultrasensitive and mutation-resilient detection of the SARS-CoV-2 nucleocapsid protein
Source: Genes Dis. 2025 Nov 19;13(3):101943. doi: 10.1016/j.gendis.2025.101943 (PMC12886537; doi:10.1016/j.gendis.2025.101943)
Supplement: Multimedia component 1 [file mmc1.docx]

**
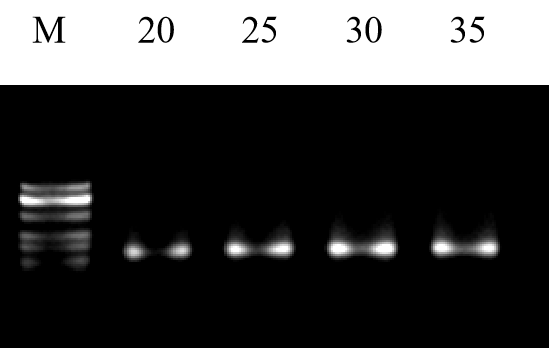
**

**Figure S1.** Using a 12% PAGE gel, 5 μL of PCR products from different cycle numbers (20, 25, 30, and 35 cycles) and 5 μL of nucleic acid dye was used to optimize the PCR cycle number.


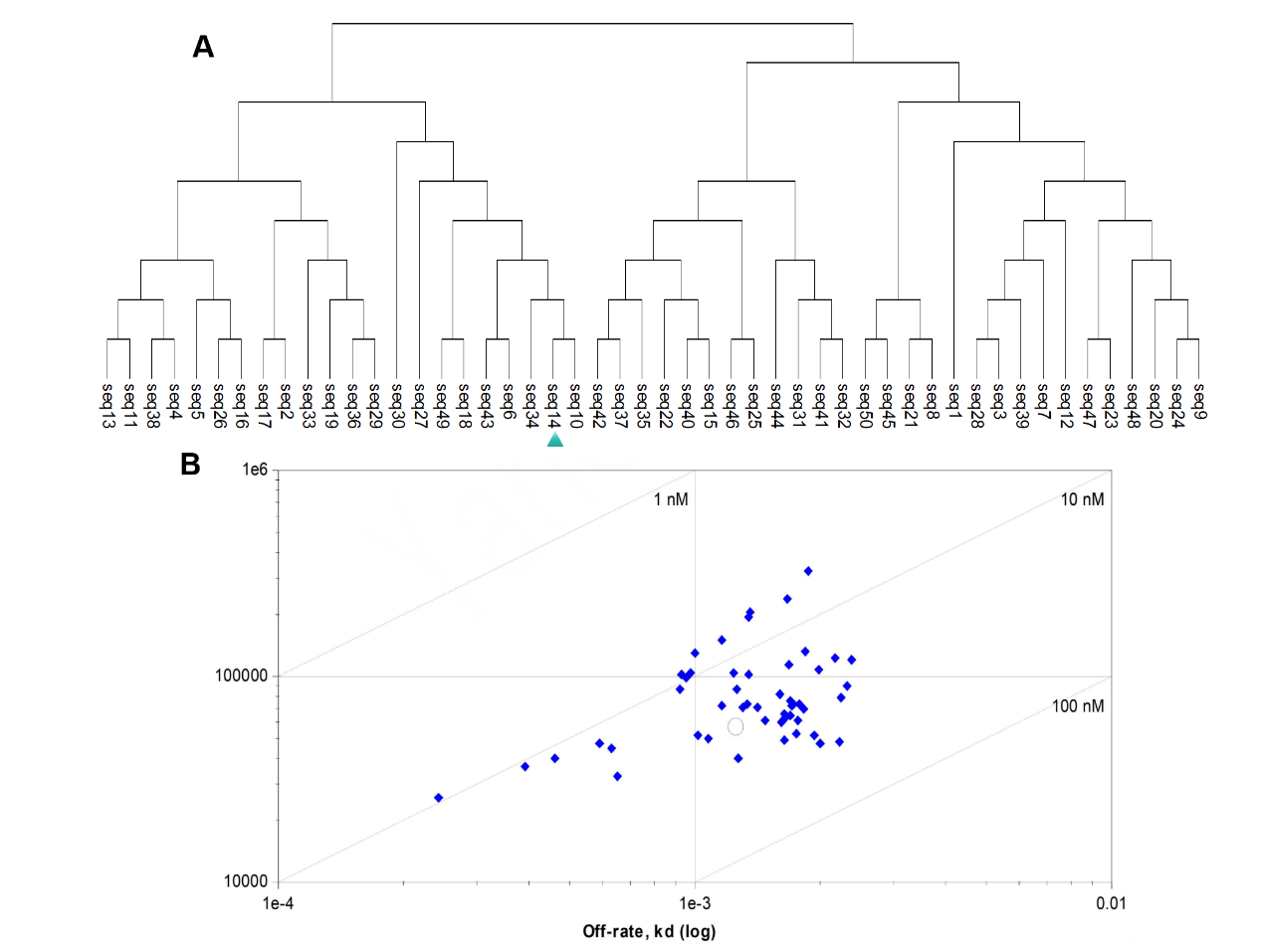


**Figure S2.** Homology analysis of the top 50 sequences. (A) Constructing a phylogenetic tree for the first 50 nucleic acid sequences. (The green triangle indicates the sequences selected for further analysis). (B) Single-concentration SPR distribution of the first 50 nucleic acid sequences.
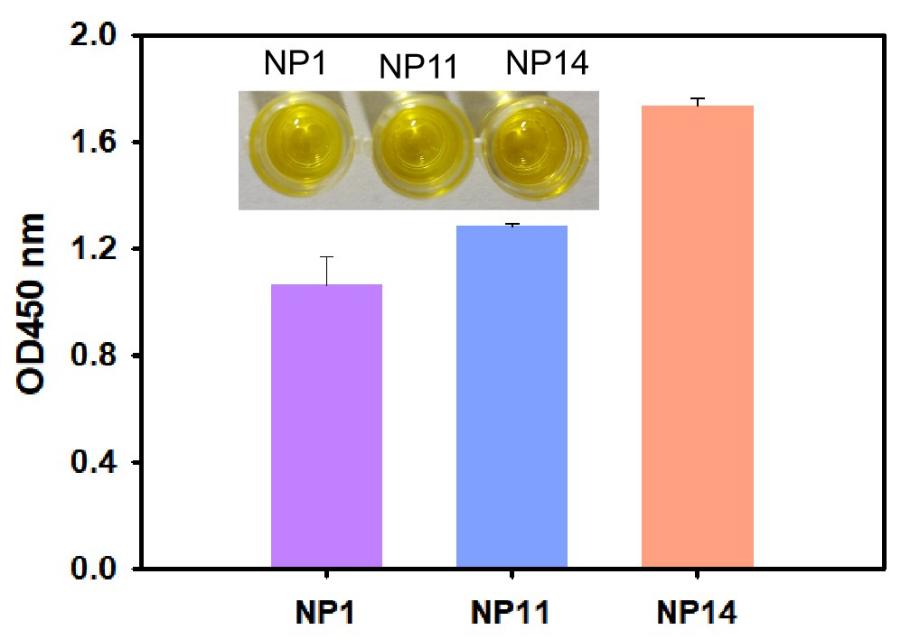


**Figure S3.** ELONA experiments validated the binding of NP1, NP11, and NP14 at a concentration of 300 nM to the SARS-CoV-2 N protein at a concentration of 1.5 μg/mL, with NP14 exhibiting a superior binding activity. Data are presented as the mean ± s.d. of four replicate results (n=4).

**
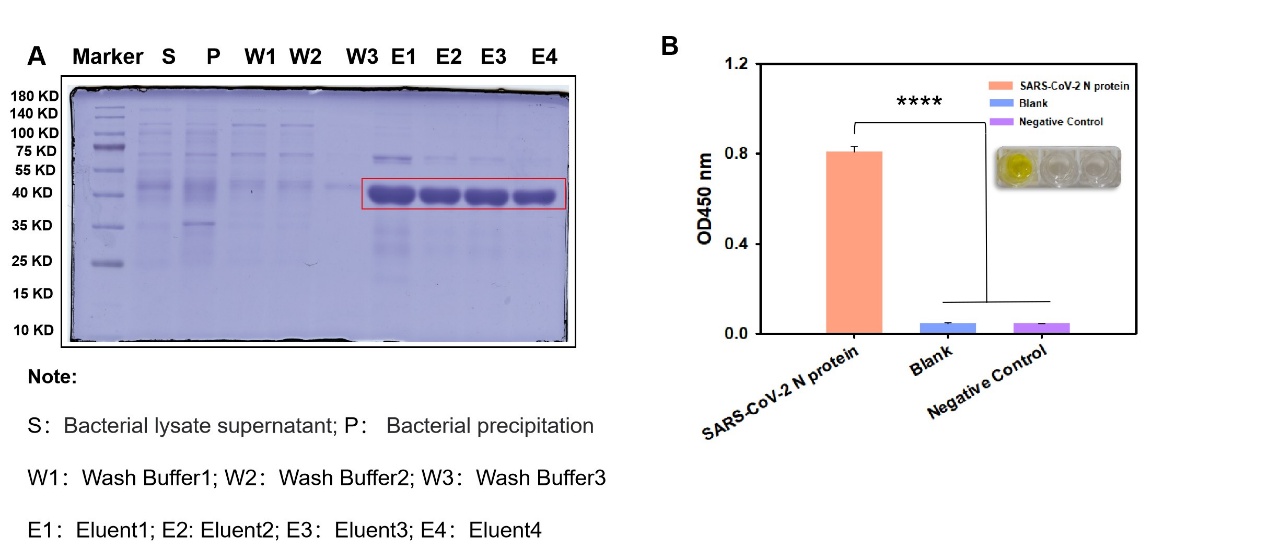
**

**Figure S4.** (A) The lysate supernatant, precipitate, wash buffers, and elution buffers (20 μg per lane) were analyzed by 10% SDS-PAGE electrophoresis during the purification process. (B) The immunogenicity of SARS-CoV-2 N protein was evaluated using the ELISA method (1.0 μg/mL anti-SARS-CoV-2 N monoclonal antibody). Data are presented as the mean ± s.d. of four replicate results (n=4). Compared with the blank control: *****p* < 0.0001.


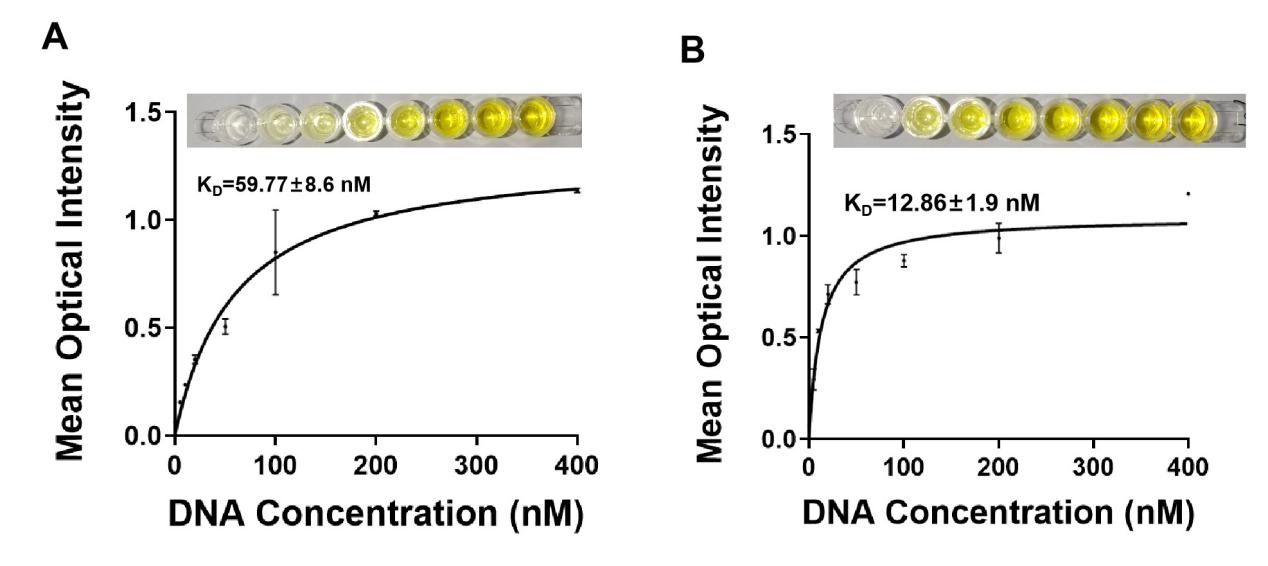


**Figure S5.** (A) The binding affinity of aptamer NP1 to 2 μg/mL SARS-CoV-2 N protein was validated and its binding properties were characterized at different concentrations (0, 5, 10, 20, 50, 100, 200, and 400 nM). (B) The ELONA method was used to validate the binding affinity of aptamer NP11 to 2 μg/mL SARS-CoV-2 N protein and at different concentrations of aptamer NP11 (0, 5, 10, 20, 50, 100, 200, and 400 nM). Data are presented as the mean ± s.d. of triplicate results (n=3).


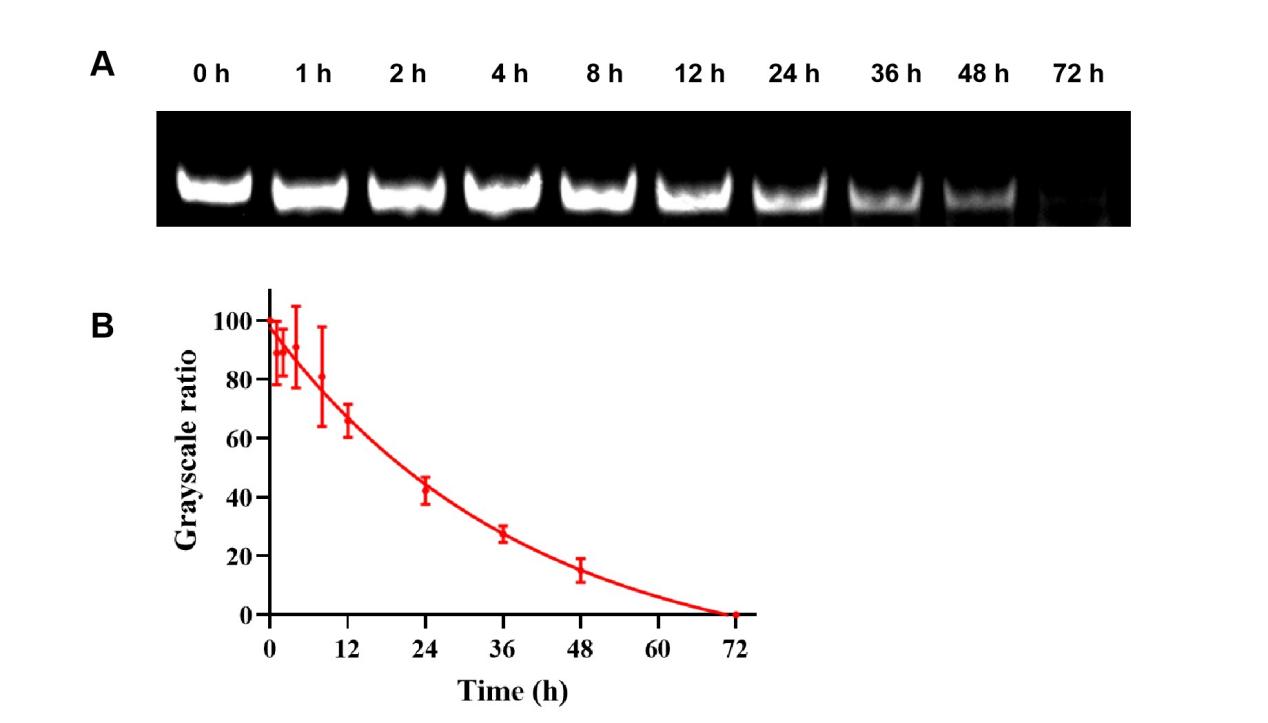


**Figure S6.** Serum stability of NP14. (A) FAM-labeled NP14 (3 μM) incubated in 10% FBS at 37 °C for 0–72 h, analyzed by 12% PAGE gel. (B) Degradation curve showing NP14 half-life extending to ~18 h. Data are presented as the mean ± s.d. of triplicate results (n=3).


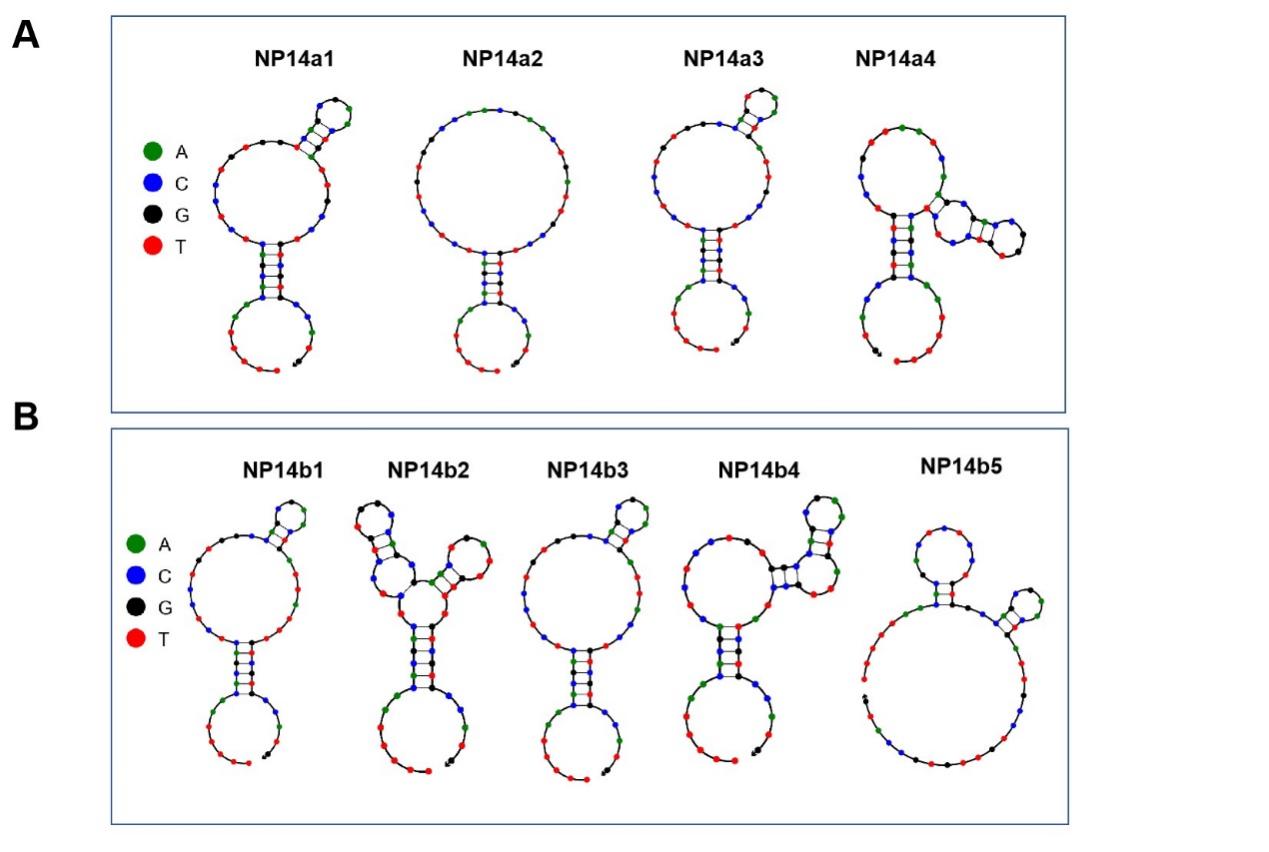


**Figure S7.** Secondary structure of the NP14 base mutant aptamer via the Nupack web server at 37 ℃.


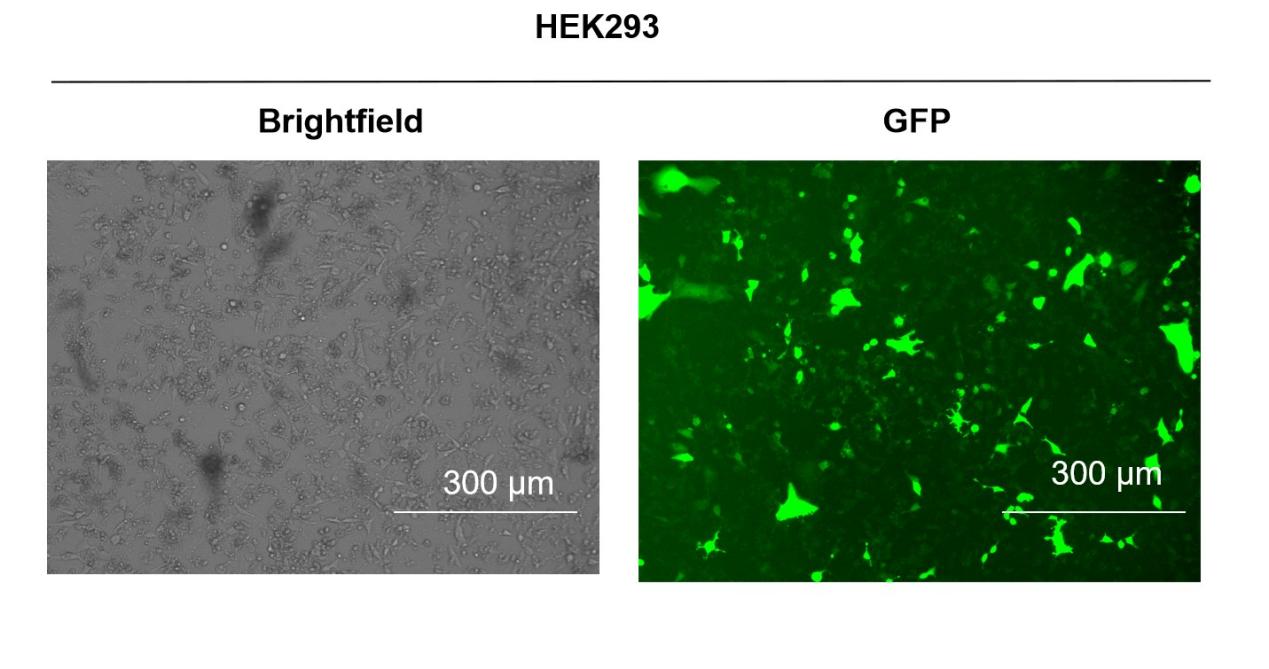


**Figure S8.** Observation of Flag SARS-CoV-2-N1-ZS Green protein expression in HEK293 cells co-transfected with 24 μL Lipo8000 and 15 μg plasmid for 48 hours via fluorescence microscopy. (N1: AA 1-219).

**
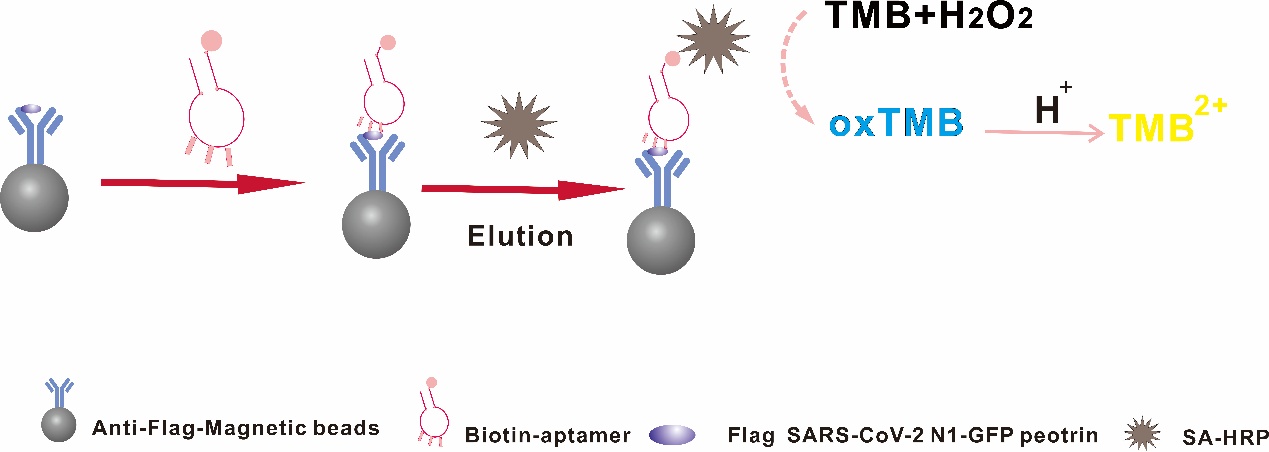
**

**Figure S9.** A schematic diagram illustrating the recognition pattern of NP14 to the SARS-CoV-2 N1 ZS Green protein was validated via immunomagnetic bead chemistry technology using immunomagnetic bead–based chemistry.


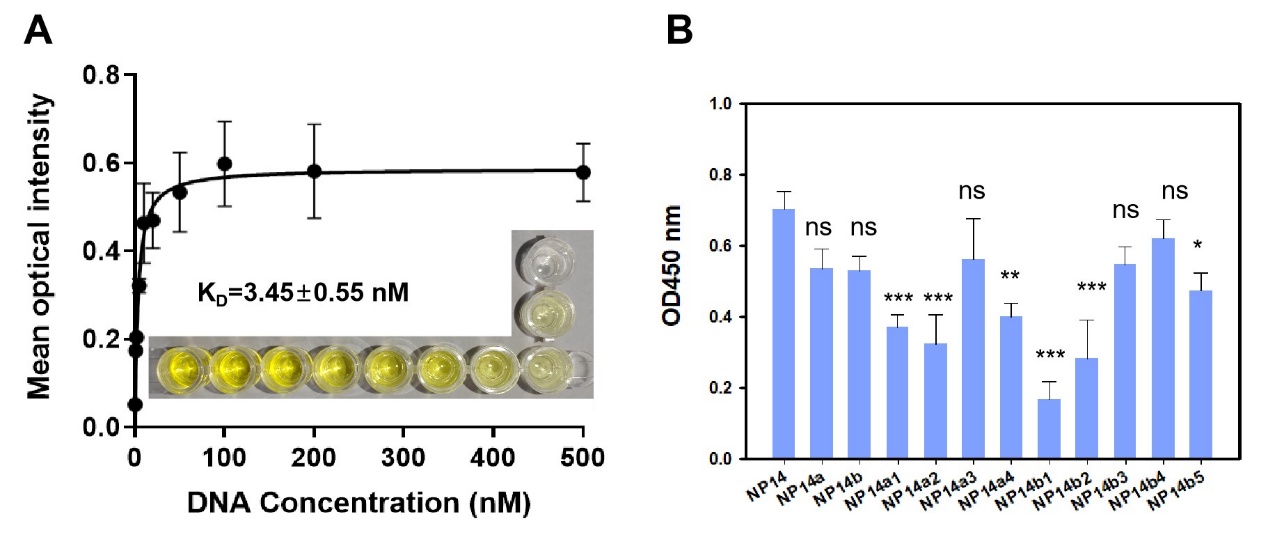


**Figure S10.** (A) The binding affinity of NP14 to SARS-CoV N protein was validated using 2 μg/mL SARS-CoV N protein and NP14 at different concentrations (0, 1, 2, 5, 10, 20, 50, 100, 200, and 500 nM). Data are presented as the mean ± s.d. of four replicate results (n=4). (B) Binding analysis of 400 nM NP14 with 400 nM truncated NP14a, NP14b, and base-mutated 400 nM NP14a1, NP14a2, NP14a3, NP14a4, NP14b1, NP14b2, NP14b3, NP14b4, and NP14b5 to the 2 μg/mL SARS-CoV N protein by ELONA. Data are presented as the mean ± s.d. of triplicate results (n=3). Compared with the NP14: ns, not significant; **p* < 0.05; ***p* < 0.01; ****p* < 0.001.


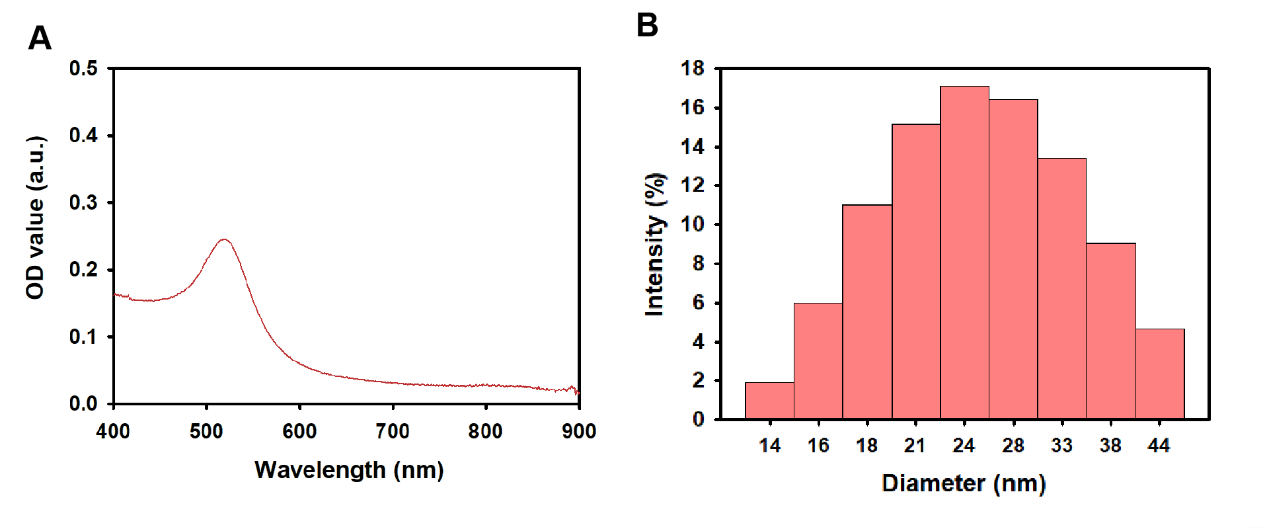


**Figure S11.** Characterization of seed gold. (A) UV-visible spectrum with absorption peak at 520 nm. (B) Hydrodynamic size distribution: Z-average 30 nm, number mean 18 nm, PDI = 0.194 (good dispersion).


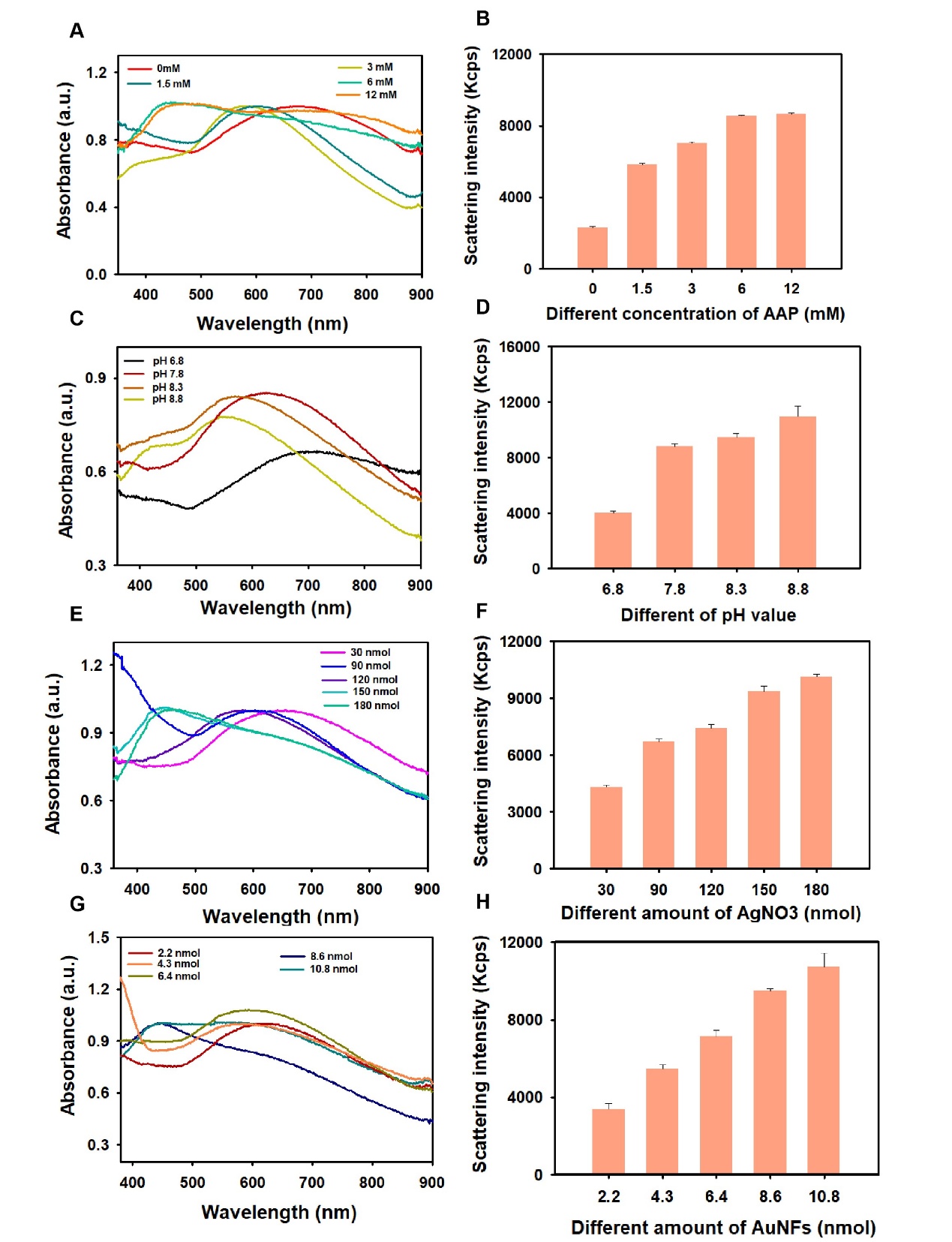


**Figure S12.** Optimization of the experimental parameters. (A) The effect of different concentrations of AAP (0, 1.5, 3, 6 and 12 mM) was characterized using ultraviolet spectrophotometry. (B) The effect of different concentration of AAP (0, 1.5, 3, 6, and 12 mM) was characterized using a particle size analyzer. (C) The effect of different Tris-HCl buffer pH values (6.8, 7.8, 8.3, and 8.8) was characterized using ultraviolet spectrophotometry. (D) The effect of different Tris-HCl buffer pH values (6.8, 7.8, 8.3, and 8.8) was characterized using a particle size analyzer. (E) The amount of AgNO₃ (30-180 nmol) was optimized and characterized using ultraviolet spectrophotometry. (F) The amount of AgNO₃ (30-180 nmol) was optimized and characterized using a particle size analyzer. (G) Effect of the amount of AuNFs (2.2-10.8 nmol) was characterized using ultraviolet spectrophotometry. (H) Effect of the amount of AuNFs (2.2-10.8 nmol) was characterized using a particle size analyzer. Data are presented as the mean ± s.d. of triplicate results (n=3).


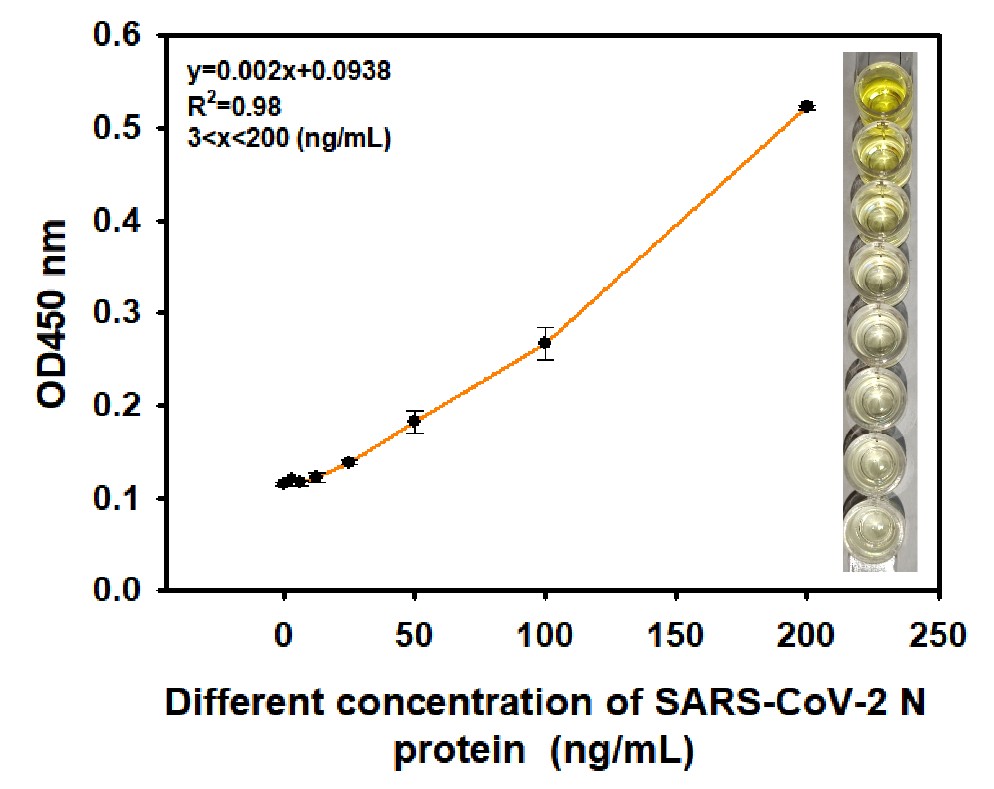


**Figure S13.** Commercial ELISA kits detect different concentrations of SARS-CoV-2 N protein (0, 3, 6,12.5, 25, 50, 100, and 200 ng/mL). Data are presented as the mean ± s.d. of triplicate results (n=3).


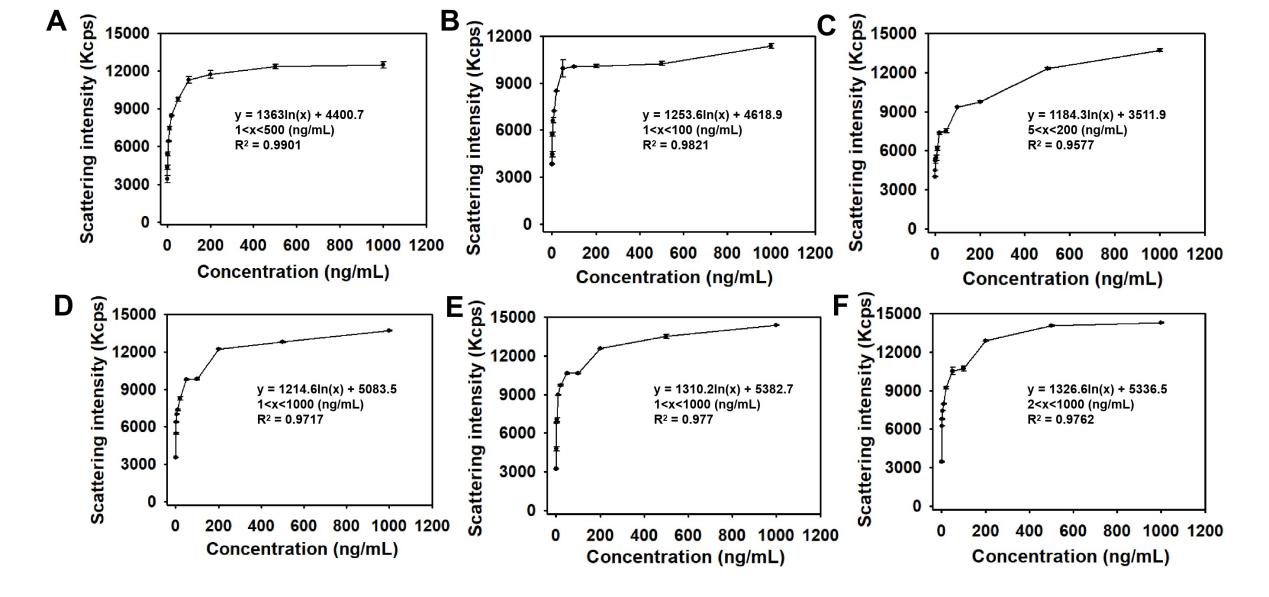


**Figure S14.** Detection of SARS-CoV-2 variants using the MD ELAAA platform. NP14 was tested for binding to recombinant nucleocapsid proteins from multiple SARS-CoV-2 variants across a concentration range of 0-1000 ng/mL. Panels: (A) Alpha, (B) Beta, (C) Gamma, (D) Delta, (E) Omicron BA.1, (F) Lambda. Data are presented as the mean ± s.d. of triplicate results (n=3).

**Table S1**. DNA sequences used in this study. (N represents the 30 random intervals in the middle.)

| Name | Sequence (5’ → 3’) | Length (nt) |
| --- | --- | --- |
| Library | TTTTTAACACG -30 N- GTCGTGCCATG | 52 |
| NP1 | TTTTTAACACGACCTCACTGTGCCGGCCACAGCTCACCACAGTGGGCCCATG | 52 |
| NP11 | TTTTTAAGCCCACTTTTCTGTGCCGGGCACAGCATGGGCTTGTCGTGCCATG | 52 |
| NP14 | TTTTTAACACGACTCTCCTGTGGCCAGCGAACTGATTGCCTGTCGTGCCATG | 52 |
| NP14a | TTTTTAACACGACTCTCCTGTGGCATTGCCTGTCGTGCCATG | 42 |
| NP14a1 | TTTTTAACACGACTCTCCTGTGGTCAGCGAACTGATTGCCTGTCGTGCCATG | 52 |
| NP14a2 | TTTTTAACACGACTCTCCTGTGGCCAACGAACTGATTGCCTGTCGTGCCATG | 52 |
| NP14a3 | TTTTTAACACGACTCTCCTGTGGCCAGTGAACTGATTGCCTGTCGTGCCATG | 52 |
| NP14a4 | TTTTTAACACGACTCTCCTGTGGCCAGCGAACTAATTGCCTGTCGTGCCATG | 52 |
| NP14b | TTTTTAACACGACTCTCCTGTGGCCAGCGAACTGATGTGCCATG | 44 |
| NP14b1 | TTTTTAACACGACTCTCCTGTGGCCAGCGAACTGATTATTTGTCGTGCCATG | 52 |
| NP14b2 | TTTTTAACACGACTCTCCTGTGGCCAGCGAACTGATTGTTTGTCGTGCCATG | 52 |
| NP14b3 | TTTTTAACACGACTCTCCTGTGGCCAGCGAACTGATTACCTGTCGTGCCATG | 52 |
| NP14b4 | TTTTTAACACGACTCTCCTGTGGCCAGCGAACTGATTGCCTATCGTGCCATG | 52 |
| NP14b5 | TTTTTAACACGACTCTCCTGTGGCCAGCGAACTGATTGCCTGTTGTGCCATG | 52 |
|  |  |  |

**Table S2.** Checkerboard optimization of mAb and NP14 concentrations. Bold entries indicate the optimized experimental conditions.

| No. | NP14 (nM) | mAbs (μg/mL) | SARS-CoV-2 N protein  OD value | Blank  OD value | P/N |
| --- | --- | --- | --- | --- | --- |
| 1 | 20 | 1 | 1.10±0.014 | 0.05±0.004 | 21.23±2.124 |
| 2 | 20 | 0.50 | 0.77±0.166 | 0.05±0.004 | 14.69±2.743 |
| 3 | 20 | 0.25 | 0.13±0.002 | 0.05±0.002 | 1.73±1.231 |
| 4 | 100 | 1 | 1.11±0.022 | 0.05±0.001 | 20.60±0.680 |
| 5 | 100 | 0.50 | 0.68±0.005 | 0.06±0.003 | 12.28±0.512 |
| 6 | 100 | 0.25 | 0.27±0.004 | 0.05±0.001 | 5.07±0.104 |
| **7** | **200** | **1** | **1.66±0.176** | **0.06±0.003** | **29.26±1.547** |
| 8 | 200 | 0.50 | 0.91±0.163 | 0.06±0.002 | 14.97±2.657 |
| 9 | 200 | 0.25 | 0.43±0.010 | 0.06±0.002 | 7.21±0.120 |

**Table S3.** Representative aptamers developed against the SARS-CoV-2 N protein

| **Aptamer name** | **Method for aptamer selection** | **Length**  **(nt)** | **Binding site** | **Sequence** | **Ref.** |
| --- | --- | --- | --- | --- | --- |
| N1 | Bead-  based SELEX | 88 | Non-NTD | GCAATGGTACGGTACTTCCGGATGCGGAAACTGGCTAATTGGTGAGGCTGGGGCGGTCGTGCAGCAAAAGTGCACGCTACTTTGCTAA | ^1^ |
| A58 | Magnetic bead-based SELEX | 58 | NTD | GCTGGATGTCACCGGATTGTCGGACATCGGATTGTCTGAGTCATATGACACATCCAGC | ^2,3^ |
| A61 | Magnetic bead-based SELEX | 58 | NTD | GCTGGATGTTGACCTTTACAGATCGGATTCTGTGGGGCGTTAAACTGACACATCCAGC | ^2,3^ |

**Table S4.** Comparative aptamer-based detection methods for SARS-CoV-2 N protein. The MD ELAAA method in this study achieved an LOD of 0.43 TCID₅₀/mL (virus cultures), demonstrating superior sensitivity.

| Method | Signal output | LOD | Ref. |
| --- | --- | --- | --- |
| nLC-MS/MS | m/z | 4.5 nM | ^4^ |
| **Solid-phase extraction capillary electrophoresis-mass spectrometry** | m/z | 0.5 μg/mL | ^5^ |
| CRISPR electrochemical | electrochemical signal | 8.18 nM | ^6^ |
| A dual-aptamer electrochemical biosensor | electrochemical signal | **8.33 pg/mL** | ^7^ |
| CRISPR/Cas12a-Derived electrochemical aptasensor | electrochemical signal | 16.5 pg/mL | ^8^ |
| Glass Micropipettes | electrochemical signal | 2.26 fg/mL | ^9^ |
| Tyramide Signal Amplification | fluorescence | **48.9 ng/mL** | ^10^ |
| A single-molecule counting platform | fluorescence | 0.84 ag/mL | ^11^ |
| **Time-resolved fluorescence immunoassay** | fluorescence | **203.78 pg/mL** | ^12^ |
| Chemiluminescence | ECL | 0.01 ng/mL | ^13^ |
| Beads-fluorescence | fluorescence | 150 fg/mL | ^14^ |
| Fluorescence analysis | fluorescence | 1.0 pg/mL | ^15^ |
| Structure-switching aptamer | fluorescence | 1.0 PFU/assay | ^16^ |
| **HCR** | **glucose** | **1 pg/mL** | ^17^ |
| LFA | fluorescence | 1.427 **pg/mL protein**  1643 U/mL pseudovirion | ^18^ |
| Digital ELISA | fluorescence | 33.28 pg/mL | ^19^ |
| LFA | AuNPs | 2.89 pg/mL | ^20^ |
| MD ELAAA | Multicolor  Dynamic Light Scattering Intensity | 0.43 TCID_50_/mL | Our work |

References

1. Cho S-J, Woo H-M, Kim K-S, Oh J-W, Jeong Y-J. Novel system for detecting SARS coronavirus nucleocapsid protein using an ssDNA aptamer. *J Biosci Bioeng.* 2011;112(6):535-540.

2. Zhang L, Fang X, Liu X, et al. Discovery of sandwich type COVID-19 nucleocapsid protein DNA aptamers. *Chem. Commun*. 2020;56(70):10235-10238.

3. Esler Morgan A, Belica Christopher A, Rollie Joseph A, et al. A compact stem-loop DNA aptamer targets a uracil-binding pocket in the SARS-CoV-2 nucleocapsid RNA-binding domain. *Nucleic Acids Res.* 2024;52(21):13138-13151.

4. Poolsup S, Zaripov E, Hüttmann N, et al. Discovery of DNA aptamers targeting SARS-CoV-2 nucleocapsid protein and protein-binding epitopes for label-free COVID-19 diagnostics. *Molecular Therapy - Nucleic Acids.* 2023;31:731-743.

5. Salim H, Pont L, Giménez E, Poolsup S, Berezovski MV, Benavente F. On-line aptamer affinity solid-phase extraction capillary electrophoresis-mass spectrometry for the determination of SARS-CoV-2 nucleocapsid protein. *Microchem J.* 2025;208:112505.

6. Lo Y, Siu RHP, Tran C, Jesky RG, Kinghorn AB, Tanner JA. An aptamer/CRISPR electrochemical (ACE) biosensor for Plasmodium falciparum histidine-rich protein II and SARS-CoV-2 nucleocapsid protein. *Microchem J.* 2025;212:113176.

7. Ramanathan S, Gopinath SCB, Ismail ZH, Md Arshad MK, Poopalan P. Aptasensing nucleocapsid protein on nanodiamond assembled gold interdigitated electrodes for impedimetric SARS-CoV-2 infectious disease assessment. *Biosens. Bioelectron.* 2022;197:113735.

8. Han C, Li W, Li Q, et al. CRISPR/Cas12a-Derived electrochemical aptasensor for ultrasensitive detection of COVID-19 nucleocapsid protein. *Biosens. Bioelectron.* 2022;200:113922.

9. Tang H, Zhang S, Yang B, Qiu X, Wang H, Li Y. Metal–Organic Framework Sub-Nanochannels within the Confined Micropipettes: Precise Construction Makes It a Universal Aptamer-Based Sensing Platform. *Anal. Chem.* 2024;96(44):17649-17656.

10. Huang Z, Du Z, Li J, et al. Aptamer-Based Activatable Tyramide Signal Amplification for Low-Background Detection of SARS-CoV-2 Nucleocapsid Protein. *Anal. Chem.* 2025;97(1):328-336.

11. Zhu J, Zhao X, Mao J, Na N, Ouyang J. Single-Molecule Evaluation of the SARS-CoV-2 Nucleocapsid Protein Using Gold Particle-in-a-Frame Nanostructures Enhanced Fluorescent Assay. *Anal. Chem.* 2023;95(12):5267-5274.

12. Chen H, Liu T, Zhao X, et al. Development of a Peptide Aptamer-Based TRFIA for the Quantitive Detection of SARS-CoV-2 Nucleocapsid Protein. *J. Fluoresc.* 2025.

13. Chen Z, Wu Q, Chen J, Ni X, Dai J. A DNA Aptamer Based Method for Detection of SARS-CoV-2 Nucleocapsid Protein. *Virol. Sin.* 2020;35(3):351-354.

14. Liu J, Mao J, Hou M, Hu Z, Sun G, Zhang S. A Rapid SARS-CoV-2 Nucleocapsid Protein Profiling Assay with High Sensitivity Comparable to Nucleic Acid Detection. *Anal. Chem.* 2022;94(42):14627-14634.

15. Zhou C, Lin C, Hu Y, et al. Sensitive fluorescence biosensor for SARS-CoV-2 nucleocapsid protein detection in cold-chain food products based on DNA circuit and g-CNQDs@Zn-MOF. *LWT.* 2022;169:114032.

16. Lim J, Son SU, Ki J, et al. Dual structure-switching aptamer-mediated signal amplification cascade for SARS-CoV-2 detection. *Biosens. Bioelectron.* 2024;259:116375.

17. Yin W, Hu J, Chen F, et al. Combining hybrid nanoflowers with hybridization chain reaction for highly sensitive detection of SARS-CoV-2 nucleocapsid protein.  *Anal. Chim. Acta* 2023;1279:341838.

18. Li H, Fu X, You Q, et al. Multiple aptamer recognition-based quantum dot lateral flow platform: ultrasensitive point-of-care testing of respiratory infectious diseases. *J. Mater. Chem. A.* 2025;13(5):1681-1689.

19. Ge C, Feng J, Zhang J, et al. Aptamer/antibody sandwich method for digital detection of SARS-CoV2 nucleocapsid protein. *Talanta.* 2022;236:122847.

20. Kim J, Baek S, Nam J, et al. Simultaneous Detection of Infectious Diseases Using Aptamer-Conjugated Gold Nanoparticles in the Lateral Flow Immunoassay-Based Signal Amplification Platform. *Anal. Chem.* 2024;96(4):1725-1732.
